# A scalable security co-processor design for IoT applications

**DOI:** 10.1038/s41598-026-58079-9

**Published:** 2026-06-21

**Authors:** Mohamed Niazy, Haytham Azmi, Mervat M. A. Mahmoud

**Affiliations:** Microelectronics Department, Electronics Research Institute, Jozeph Tito, New Nozha, Cairo, 11843 Egypt

**Keywords:** Application-specific instruction-set processor (ASIP), Cipher-security, IoT, Encryption, RISC-V, Direct memory access (DMA), Engineering, Mathematics and computing

## Abstract

The development and implementation of scalable and efficient security solutions have become a critical area of focus in cybersecurity research and practice. This trend is receiving more attention lately due to the increasing frequency and sophistication of cyber threats to Internet of Things (IoT) networks. This paper proposes a novel cryptographic co-processor architecture based on RISC-V, designed to offer both high efficiency and flexibility for IoT applications. The design introduces a generic interface for cipher blocks, supports parallel execution of cipher operations, and avoids custom modifications to the RISC-V instruction set architecture. The proposed design extends the RISC-V architecture without new instruction sets to support AES-128 and SHA-256 operations, demonstrating its capabilities through these ciphering blocks. The new processor architecture ensures efficient data transfers between memory and cipher units, without delaying the processor pipeline, by utilizing Memory-Mapped I/O (MMIO) and Direct Memory Access (DMA) modules to optimize data handling. The proposed design is implemented and verified on the Xilinx ZCU-102 FPGA board using the Vivado 2022.2 tool. The proposed design achieves throughput rates of 8.2 Gbps for the AES-128 cipher block and 482 Mbps for the SHA-256 cipher block, operating at a relatively low system clock frequency of 64 MHz. The throughput is calculated based on the core cycle count of each algorithm, as this metric is commonly adopted in the literature. Nevertheless, the total end-to-end cycles of the proposed design equal the core cycles plus the serializer and deserializer cycles. The cycle count of the serializer/deserializer depends only on the processor’s data bus width. Also, the total power consumption of the proposed design is 1.575 watts. Achieving such high throughput at this reduced frequency is significant as it helps designers better minimize the power consumption, thereby enhancing the overall energy efficiency and performance of the system.

## Introduction

As the complexity and frequency of cyber threats in Internet of Things (IoT) applications continue to rise, researchers have begun to explore advanced security solutions to meet the growing demands of the IoT industry. Security-specific processors, which offload cryptographic and other security-related tasks from the main processor to a tailor-built processor, play a vital role in protecting sensitive data in IoT systems^1,2^. Data integrity and confidentiality between IoT nodes should be guaranteed by lightweight cryptographic techniques to access data and pass all security validations in real time^3^. Recent surveys recommend security-specific processors in IoT systems, Radio Frequency Identification (RFID), and digital signatures. However, traditional security-specific processor designs often face limitations in scalability and flexibility, making it difficult to adapt to evolving security requirements^4,5^. A reconfigurable and scalable architecture for security co-processors is needed to address such challenges.

In the evolving landscape of computer architecture, the RISC-V instruction set architecture (ISA) has received significant attention due to its flexibility, extensibility, and open source nature^6^. However, a significant challenge in extending the functionalities of the RISC-V to act as a cryptography co-processor is the capability to handle data of varying sizes that manage different cryptographic operations. Additionally, the on-chip communication interfaces that connect the Central Processing Unit (CPU) to the peripheral cryptography algorithms have several I/O mapping methods.

Creating a cryptography instruction set to control the cipher blocks and then programming the control unit to handle their opcode has some disadvantages. The cipher algorithms require several clock cycles, which stall the processor for many clock cycles. Additionally, the new instructions do not make the design fully compatible with the RISC-V standard and its compilers or libraries. Numerous microcontrollers use isolated registers for controlling peripherals. It involves mapping I/O devices to a specific set of registers within the CPU or peripheral module. These registers act as control points for communication with external hardware. The CPU reads and writes from these registers to control the devices directly. On the other hand, memory access for I/O is more suitable for cryptography algorithms as it maps the input/output devices to a portion of the memory address space. DMA with Memory-Mapped I/O (MMIO) maps I/O data to the system memory address space, allowing the CPU to interact with the hardware using regular memory access instructions. The devices appear as part of the system memory, making it simpler to manage and access^7^.

This research work aims to address these design challenges by proposing a new co-processor architecture design based on RISC-V that can be extended to handle various cryptographic operations in IoT systems.

This paper has the following main contributions:

- *Novelty*: Proposed a novel cryptographic co-processor architecture for IoT applications based on RISC-V, without new instruction sets to support AES-128 and SHA-256 operations.
- *Scalability*: Designed a generic interface for the extended cipher blocks, which allows additional encryption/decryption algorithms to be added easily to the RISC-V architecture in IoT edge devices.
- *Performance optimization*: The new processor architecture ensures efficient data transfers between memory and cipher units, without delaying or stalling the processor pipeline, by utilizing Memory-Mapped I/O (MMIO) and Direct Memory Access (DMA) modules to optimize data handling.
- *Testing and verification*: The proposed design is implemented and verified on the ZCU-102 FPGA board and achieves throughput rates of 8.2 Gbps for the AES-128 cipher block and 482 Mbps for the SHA-256 cipher block, operating at a relatively low system clock frequency of 64 MHz.

This paper is organized as follows. Section "Literature review" highlights similar work in the literature. Section "Proposed co-processor architecture" presents our proposed architecture for the security co-processor design. This includes discussing the techniques for accessing, managing, and optimizing data flow through cipher blocks within the RISC-V RI32 system. Section "Results and discussion" shows the results of the hardware implementation of the coprocessor and analyzes its performance. Finally, we draw our conclusion and suggest new research directions for future work in Sect. "Conclusion".

## Literature review

Security-oriented application specific instruction-set processors (ASIPs) are built in the literature with specific hardware units that facilitate one or more complex cryptography operations like AES, hashing functions, and the RSA algorithm. Firstly, we will start with the state-of-the-art security ASIPs for one cryptography algorithm, then the broad ASIPs for multiple cryptography operations. Following that, other designs like system-on-chip (SoC) and co-processors based on well-known processors will be discussed.

Custom AES ASIP was implemented by Tsekoura et al.^8^, in 2010. They added vector units for memory, register file, and crypto operations to design a custom processor for the AES-CCM-32 security mode. Consequently, Shahbazi and Eshghi^9^, in 2014, proposed an ASIP for the AES algorithm. Furthermore, the intensive computation of the RSA algorithms prompted designers to implement an ASIP to speed up its operations. Wang et al. in 2012^10^ extended the instructions of the pipelined 3-stages RISC processor with specific addition/subtraction, shift and multiplyAdd instructions for their RSA co-prosessor. Moreover, cipher Hashing algorithms are used in many security applications and implementations as specific-processor^11–13^. Consequently, in 2016, Huo and Liu^14^ designed a specific processor with 7 pipeline stages for hashing operations. The pipeline passes through fetch, decode, memory, read permutation network, two execute stages, and write back, respectively. The read permutation network stage is designated for hashing operations. Recently, Mehrabani et al.^15^, in 2022, implemented a custom processor with the basic arithmetic, shift, memory, and control instructions for the SHA-3 algorithm. Specific instructions are added to effectively apply the SHA-3 algorithm, among which are memory-reference and register-reference instructions. Moreover, researchers designed an ASIP for other widely used authentication functions like physical unclonable function (PUF)^16^, RFID (radio frequency identification) system^17^ and Niederreiter cryptosystem^18^. Many other state-of-the-art was work for one cryptography operation^19,20^.

However, Many security-specific processors in the literature are designed for more than one cipher algorithm. In 2007, Lu et al.^21^ designed an ASIP with a special operation unit for the RSA and ECC algorithms. A custom processor and instruction set are presented based on the traditional 5-stage pipeline architecture. Huang et al. in 2009^22^ introduced a scalable and unified pipelined crypto-processor based on the RISC processor. An implementation of the complicated operations, such as CSA and CLA, is used to unify the architecture. Shahbazi et al.^23^ presented a cryptography processor for AES, IDEA, and MD5, with custom instructions executed at 167 MHz. The results in Ref. 23 show that an 128-bit input block is encrypted after 122 clock cycles for AES-128. The MD5 hashing algorithm requires 469 clock cycles to generate the coded outputs for a block of 512 bits. SHA-256 is slower than MD5 and produces a value of 256 bits as opposed to MD5’s 128 bits.

On the other hand, numerous authors opted to develop their specific processors based on existing processors that were well-tested and high-performing, such as MIPS and RISC processors^24^. In 2012, Ju et al.^25^ proposed a custom instruction set architecture for a hash function based on the pipeline structure of the 5-stage RISC processor. They utilized the 32-bit OR1K open-source RISC architecture with duplication in the register file, decode stage, and execute stage. Two multiplexers are used to select the inputs and outputs from and to registers. In 2016, Eissa et al.^26^ implemented a pipelined MIPS-based processor for the SHA-3 hash function. They modify the ALU (Arithmetic and Logic Unit) of the MIPS architecture to extend the instruction set. Ahmadi and Mirzaee^27^, in 2019, proposed a single-cycle MIPS-core architecture for the International Data Encryption Algorithm (IDEA). Their architecture supported general-purpose and specific instructions. They added the modulo-$2^{16}+1$ multiplication, modulo-$2^{16}$ addition, additive inverse, and multiplicative inverse arithmetic operations to the ALU operations to support the encryption and decryption of the IDEA algorithm. 19.3 MHz on Virtex-6 FPGA with encoding/decoding throughput 5.578 Mbps. Wang et al.^28^ in 2021, suggest the usage of two pipelined processors, RISC-V and a cryptoprocessor, with handshaking communication and shared data memory. The supported crypto-operations are AES, ECC, and SHA-256. Also, Gomes et al.^29^, in 2022, used two processors. They connect the AES co-processor with a RISC-V Rocket-core processor. Their AES co-processor includes the cipher operations, interface module, and control unit. Moreover, Le et al.^30^ in 2024 proposed a security co-processor design based on RISC-V with custom instructions for security functions, as they add security operations to the ALU stage. Their RVCP co-processor operates at 210 MHz and supports Salsa20, ChaCha20, and AES-128 encryption algorithms, as well as several hash functions.

The widespread use of FPGAs with integrated processors motivates researchers to introduce a hardware/software co-design for cryptography ASIPs. They utilized the built-in AMD MicroBlaze softcore or ARM processor in the FPGA kits. In 2016, Varriale et al.^31^, designed a security platform based on a system-on-chip. They integrated an ARM processor, common criteria certified smartcard, and an FPGA. Toubal et al.^32^ in 2020, implemented their wireless sensor node based on the built-in AMD MicroBlaze softcore on the Artix-7 FPGA. They connect the soft core with the cipher blocks using the built-in AXI bus. They built an C/C++ customized library to exchange data with the cipher coprocessors that run in the MicroBlaze softcore. Huynh et al.^33^, in 2020, used the built-in ARM processor and IP security blocks. They used an external memory module for the hard processor system, which is used with the Avalon bus to communicate with the blocks. Furthermore, several researchers in the literature are attracted to the high-performance hardware implementation of cryptography algorithms without mentioning their integration into a processor. Kundi et al.^34^ in 2020 proposed a unified FPGA-based hardware design of a cryptographic core that integrates AES and SHA-3 to provide multi-purpose cryptographic services. They share the design resources to decrease the area and power of the system.

Summarizing the weaknesses in the state-of-the-art, security-specific processors are limited to one cryptography algorithm or add different cryptography functions to the arithmetic unit of the processor, which requires the addition of instruction sets to support these cryptography functions. Nevertheless, the use of a single-cycle or a tailored pipeline processor architecture has an effect on the latency of the processor’s normal instructions. Furthermore, a micro-programmed control unit for the new cryptography instruction set was used in the literature^35^; however, the proposed work and most state-of-the-art implementations use a hardwired control unit, which provides a faster control unit for the processor.

This research paper proposes a novel design of a scalable co-processor that could be easily integrated into systems of varying sizes and complexities, from IoT devices to large-scale enterprise networks. This flexibility is crucial in developing a future-proof security solution that effectively addresses both current and emerging threats. Our approach in designing the security co-processor was not based on implementing custom modifications to the RISC-V ISA to take advantage of the standard RISC-V hardware design workflow. On the contrary, our approach is mainly based on extending the functionality of the RISC-V architecture. The RISC-V ISA was carefully constructed to achieve a trade-off between simplicity, performance, and efficient hardware implementation. Most of the choices made in the standard, such as instruction formats, immediate encoding, and opcode structure, result from thorough analysis and optimization. For example, compared to older architectures like MIPS, RISC-V restructured immediate field positions to reduce logic complexity and the number of multiplexers required in the data path. This architectural improvement simplifies decoding and contributes to faster and more efficient circuit designs. Furthermore, using the standard ISA allows developers to take full advantage of the RISC-V ecosystem, including open-source toolchains, compilers, and assemblers. These tools are typically built to recognize and work with the official ISA. Making changes, such as redefining opcodes or altering instruction formats, would require rewriting or replacing key parts of the toolchain, including the assembler, which introduces unnecessary complexity and maintenance overhead.

## Proposed co-processor architecture

The proposed co-processor architecture is based on the RISC-V structure^36^. It comprises the five common RISC-V stages: fetch, decode, execute, memory, and write-back. In addition, two blocks are added to handle hazards. The design supports pipelining and allows different cipher blocks to run in parallel. The new cipher blocks are integrated in the memory stage, each with specific memory addresses.

This section is organized as follows. In Sect. "Cipher blocks wrapper", we discuss (1) the modifications introduced to the memory stage of the co-processor, (2) the interface of the cipher blocks with the co-processor’s other stages, and (3) the integration of the added cipher blocks into the system. Following that, later subsections will discuss and illustrate the other co-processor stages, as well as the detection and hazard forwarding blocks. Finally, the last subsection explains the handling of the cryptographic commands in our proposed co-processor. For consistency purposes, the signal names in the figures begin with an abbreviation of their originating stage: Fetch, Decode, Execute, Memory, Write-Back, and Forwarding as IF, ID, EX, MEM, WB, and ForwardING, respectively.

### Cipher blocks wrapper

**Fig. 1:**
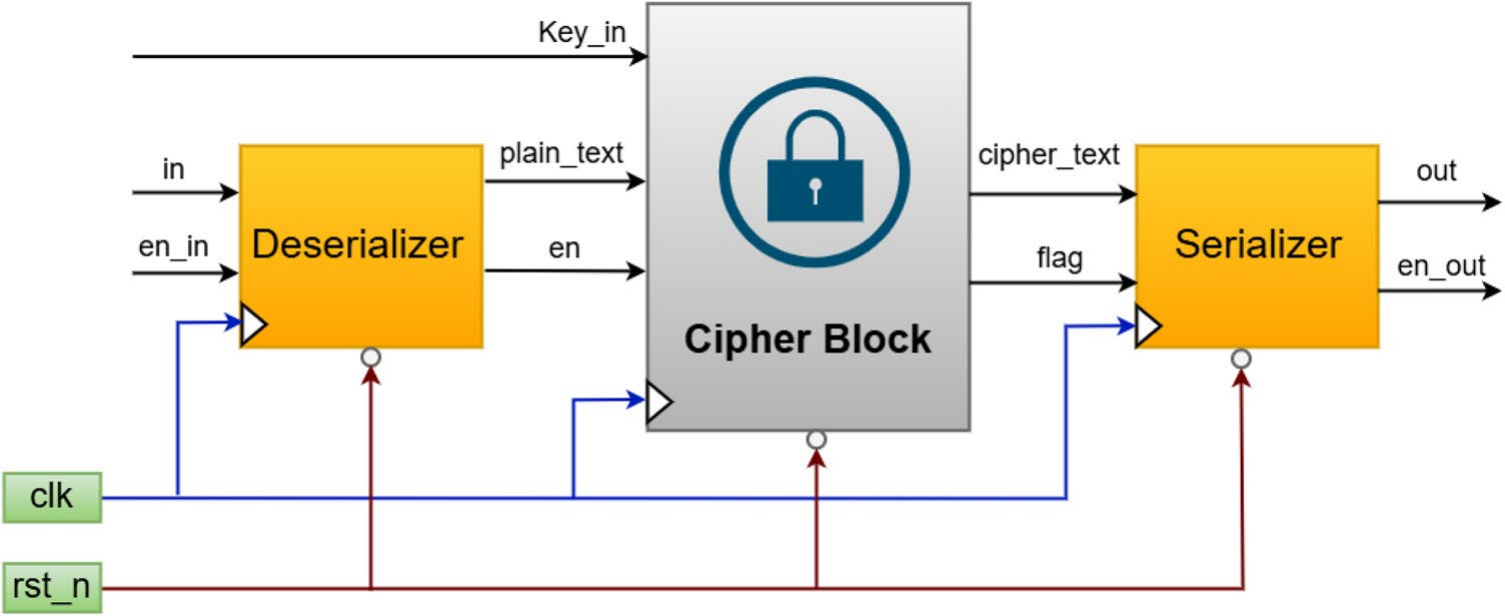
AES-128 wrapper.

**Fig. 2:**
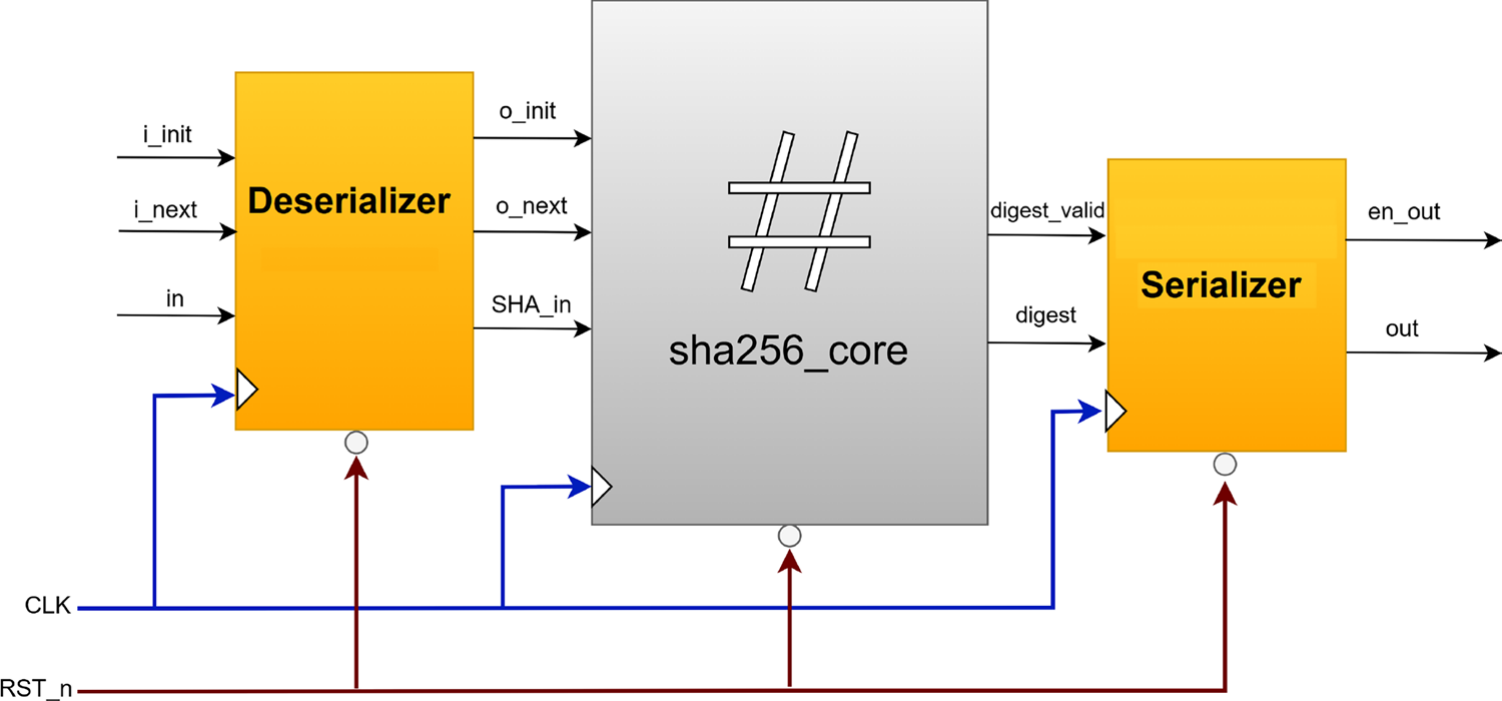
SHA-256 wrapper.

Figures 1 and 2 illustrate the suggested wrapper architecture for the cipher blocks. Cryptographic algorithms, such as hashing, AES, and RSA, typically have variable-sized plaintext inputs and generate ciphertext outputs.

The first challenge in this integration is that the system registers and memory words are fixed to a 32-bit width. However, the size of the plain text varies across different cryptographic functions. To address this, a deserializer is utilized at the input ports of the cipher block to transform serial data from the RISC-V interface into parallel data. This module takes 32-bit data inputs and, over *n* clock cycles, converts them into a (32$\times$*n*)-bit format, where *n* represents the number of words in the input data.

Similarly, a serializer is placed at the output of the cipher block to convert data from the cipher peripheral interface back into a serial format. It transforms the (32$\times$*n*)-bit output into 32-bit segments over *n* clock cycles. While this modification introduces some latency, it enables pipelining and allows the parallel execution of different cryptographic functions.

An enable signal, *en*, is included as an input to the cipher block to indicate that the input plain text is ready for processing. Additionally, an enable signal, *en_in*, is provided to the deserializer input to initiate the reading of 32 bits for *n* clock cycles.

The second challenge is that the latency is determined by the specific cipher algorithm and its implementation. Therefore, a *flag* signal is added to the output to indicate to the serializer that processing is complete.

Two fundamental cryptographic functions, AES-128 and SHA-256, are integrated into the proposed co-processor architecture. Figures 1 and 2 depict the specific wrappers for both modules, while Table 1 details their respective input/output port definitions.

**Table 1:** Input/output port definitions for AES-128 and SHA-256 integration.

Cipher block	Port name	Direction	Size (bit)	Port definition
Shared signals	clk	input	1	Clock signal
	rst	input	1	Asynchronous negative reset signal
AES-128	EN	input	1	Enable signal to start cipher processing
	plain_text	input	128	Data to be encrypted
	key_in	input	128	Encryption key
	cypher_text	output	128	Encrypted output
	flag	output	1	Status flag
SHA-256	i_init	input	1	Control signal indicating the start of a new message sequence
	i_next	input	1	Control signal indicating that the current input contains subsequent bits of the continuing message
	in	input	512	Input message block to be hashed
	en_out	output	1	Status flag
	out	output	256	Hashed output (Digest)

For the AES-128 module, the encryption command sends a pulse to the *en_in* signal. This triggers the deserializer to read four words from memory, outputting the 128-bit plain text and generating an *en* pulse to activate the AES cipher block. The AES-128 block requires 128-bit plain text and a pulse on the *EN* signal to begin encryption. Upon completion, it raises the *flag* signal high and outputs the encrypted data.

Similarly, the wrapper for the SHA-256 hashing function follows this structure. However, the *Key_in* input is not utilized. Instead, the control logic relies on the *i_init* and *i_next* signals to manage message sequencing; *i_init* indicates the start of a new message sequence, while *i_next* signifies that the current input consists of subsequent bits of a continuing message. The deserializer reads the complete input message from memory, word by word, before generating the enable pulse. The SHA-256 module outputs fixed-size data (256 bits, or eight memory words). While the hashing function requires a significant number of clock cycles to complete this secure algorithm, the co-processor remains available during this time, operating in parallel with processor instructions.

### Integration of cipher blocks with the RISC-V co-processor

This work uses a pipelined co-processor that begins cipher operations during the execution phase and thereafter detaches from the operation until its completion. The input data for the cipher block must be prepared before its beginning, and upon completion, an interrupt is sent back to the co-processor. The memory access is managed indirectly through a Direct Memory Access (DMA) module, which handles various corner cases and facilitates data transfers between cipher blocks, co-processor, and data memory. The DMA includes a Finite State Machine (FSM) for each cipher block, address decoder, and memory output multiplexer. The address decoder manages access to specific sections within the data memory and directs requests to the appropriate memory address. The memory output multiplexer selects the required data and ensures that the desired data is routed to the output.

#### Cipher block interface with data memory

The main challenge in the design is determining the appropriate location to store the input and output data of the cipher blocks, which have variable input file sizes and execution times. A dual-port data memory is implemented, with one port designated for the RISC-V and an external port for the user’s cipher input data and the storage of the cipher output data. The connections between the co-processor and cipher peripherals are implemented using the Memory-Mapped I/O (MMIO) technique. A direct memory access (DMA) module controls the data transfer between peripherals and cipher blocks, addressing multiple corner cases in memory access.

**Fig. 3:**
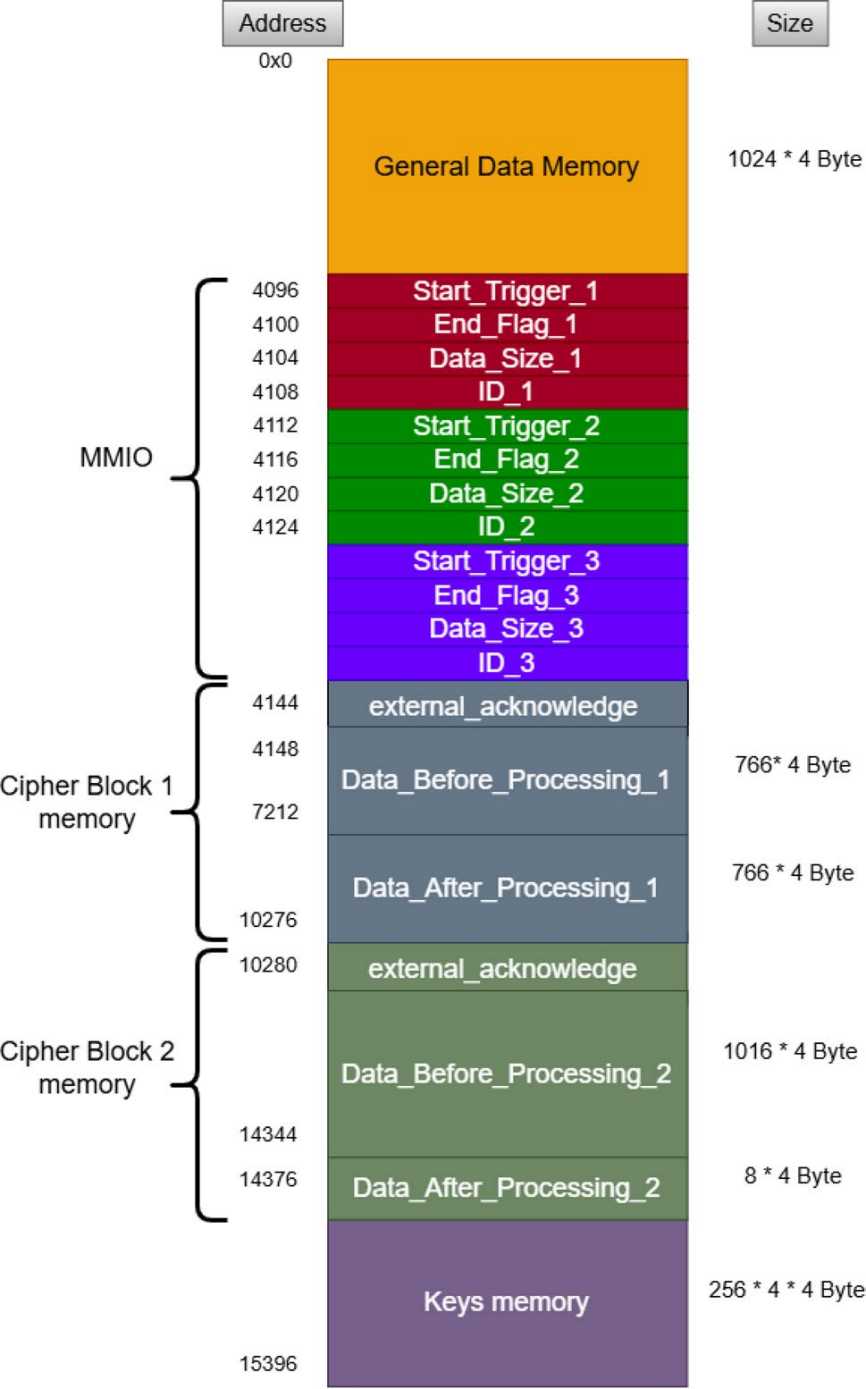
Data memory portions.

The DMA controls three memory blocks in addition to one block for each cipher algorithm. They are designed and embedded into a unified memory space with a single shared address range. The memory has a 32-bit, 4-byte, word size. The Unified Data Memory is implemented as a true dual-port structure with two independent read ports and two independent write ports. This design enables concurrent and conflict-free memory access by both the RISC-V co-processor and external users. While the RISC-V core performs processing and control operations on the cipher data, external users simultaneously store plaintext inputs and retrieve processed outputs. Providing dedicated read and write ports for each entity eliminates access contention, supports parallel data transfers, and ensures continuous operation of the cipher blocks without stalling either the processor or the external interface. Figure 3 shows the implemented data memory address ranges.

The general data memory, which serves the co-processor, has a depth of 1024 words (equivalent to 4 KB). MMIO Registers, partitioned into groups of four, are used for the cipher blocks configuration. The four registers hold the start and end flags of the cipher operation, as well as the size and ID of the cipher data. Furthermore, a cipher block memory space is used to store the input and output data of the cipher process. Its first address serves as an external acknowledgment to the RISC-V, validating the stored data that needs processing by the cipher block. The cipher block 1 and 2 memory is used for the AES and hashing algorithms. The specific memory of the AES occupies 1532 words, with an additional word reserved for control purposes, totaling 6 KB + 4 bytes. Finally, a memory space for storing keys of the cryptography operations.

Each cipher block has a DMA that manages its operations based on the I/O-mapped registers, controls the transfer and storage of data within the data memory partitions, and sets the status in the flag registers. The start flag register is accessible only by the co-processor, which writes to it through the finite state machine in DMA. When the co-processor writes a ’1’ to this register, the DMA initiates data transfer to the cipher block. The end flag register is accessible only by the DMA, used to observe the cipher blocks’ status. The file size registers, ID registers, and cipher block memory are accessible by the co-processor and the external users. The file size specifies the size of the cipher input file. The ID is used to get the private/public key of the cipher operation from the key’s memory space. The cipher block memory is duplicated for each new cipher block. Although a single memory could serve all cipher blocks, this duplicated structure allows each cipher block to operate in parallel. With separate memory instances, each memory port remains available when other blocks are busy, optimizing parallel processing. The memory space for the input and output data of cipher blocks varies depending on the cipher algorithm specs. In the case of AES, the memory is divided equally between plaintext and ciphertext. For SHA-256, most of the memory is allocated for plaintext, with only 256 bits reserved for the hashing output data. A dedicated key memory is included to store cryptographic keys required by the supported encryption algorithms. Each address in the key memory corresponds to a specific user ID and stores the associated key value. The key memory has a fixed width of 128 bits, and it is accessed in read-only mode by the DMA during cryptographic operations. External users are permitted to write or update key values only when operating in an administrative mode by providing a valid password through dedicated password input ports. The password is fixed at design time and defined during RTL implementation. During a key update operation, the DMA compares the input password against the internally stored reference password; if the comparison is successful, the new key value is written to the key memory. Otherwise, the write operation is ignored, and the existing key remains unchanged.

#### Memory stage model with AES-128 and SHA-256 cipher blocks

**Fig. 4:**
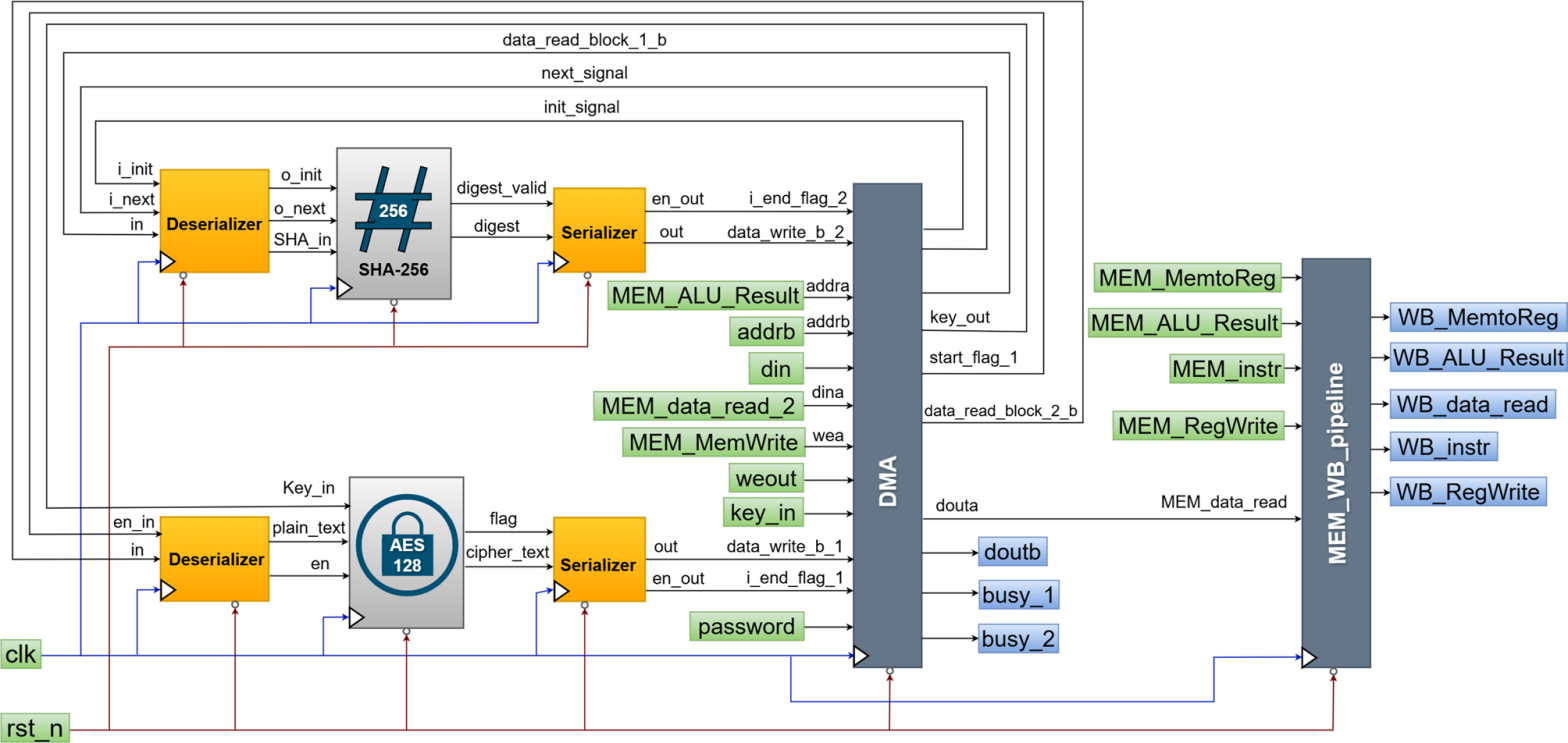
Memory stage interface with DMA and cipher peripherals.

Figure 4 shows the memory stage, including its interface with an AES encryption/decryption block and a hashing function block. The addresses and sizes mentioned in Fig. 3 specify AES-128 and SHA-256 for the cipher blocks 1 and 2, respectively. The resources required for the AES-128 operation are a serializer, a deserializer, a block of memory to store the files before and after encryption/decryption, DMA to handle data transfers between memory and AES, and an execution stage that triggers the DMA enable signal and initiates the AES operation. And the same for the hashing function.

**Fig. 5:**
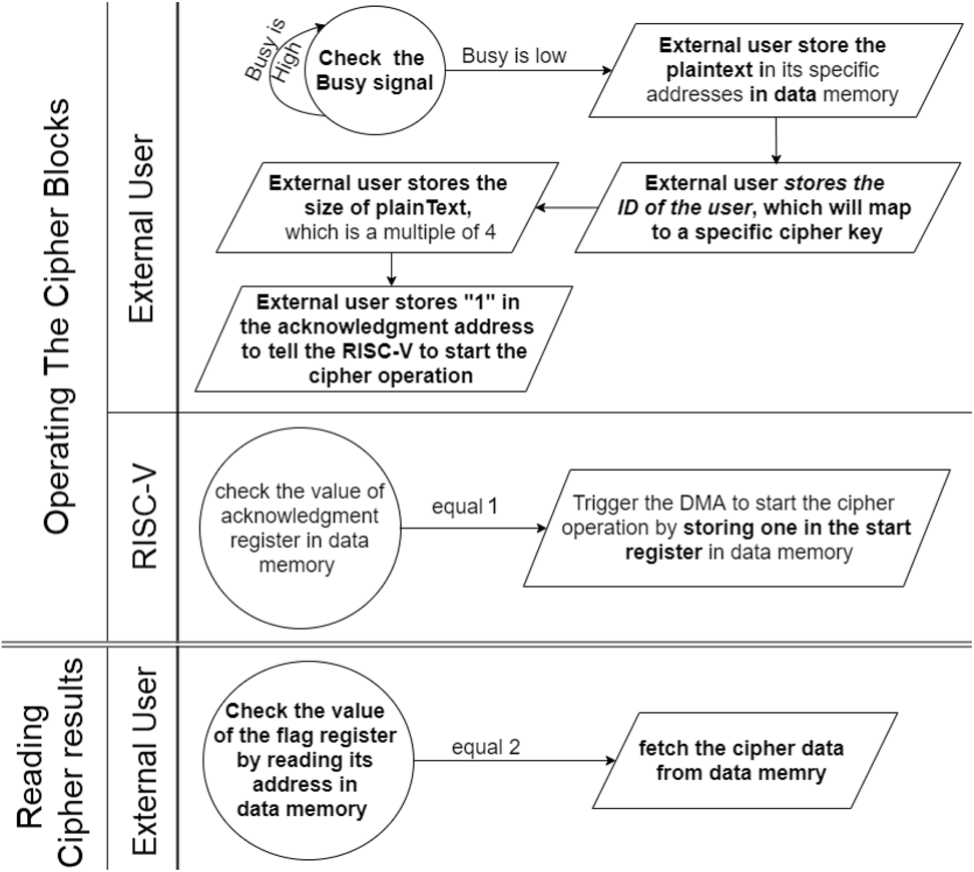
The control flow diagram between the cipher blocks and the external user and RISC-V.

The control flow between the external user and the co-processor for any cipher block is shown in Fig. 5. The RISC-V processor interacts with the cipher blocks through two operations. It verifies the acknowledgment status by reading the designated acknowledgment register in the data memory and triggers the cipher block by writing a value of one to a specific start register in data memory. For example, the steps required to perform the AES-128 encryption process. The compiler permanently checks the status of the AES by examining the $busy\_1$ flag, output from the DMA. If it is high, it indicates that AES is processing $plain\_text$ and no data can be written or read from the memory. The AES is idle when the $busy\_1$ flag is low. The DMA maps the $End\_Flag\_1$ register in the data memory and stores the AES status. It holds a value of ’1’ when cipher processing starts and ’2’ when it completes. When the $End\_Flag\_1$ register holds a value equal to ’1’, the plaintext file, ID, and file size are stored in the data memory and store ’1’ in the $start\_Trigger\_1$ to initiate the AES process. The co-processor triggers the start signal, prompts DMA to set the busy flag, and begins sending the *plaintext* to the AES. The process continues until it encrypts the entire file and stores the encrypted results. Once encryption is complete, DMA stores ’2’ in the $End\_Flag\_1$ register to indicate that the encryption process has finished.

### Basic RISC-V stages and hazard handling blocks

The RISC-V processor core used in this work was developed from scratch in Verilog by the authors. The design follows the RV32I instruction set architecture and was specifically tailored to support tight integration with the proposed cryptographic co-processor. This custom implementation enabled full control over the pipeline structure, hazard handling mechanisms, and memory-mapped interfaces, allowing seamless integration of DMA control, cipher blocks, and unified data memory without relying on external processor IPs.

**Fig. 6:**
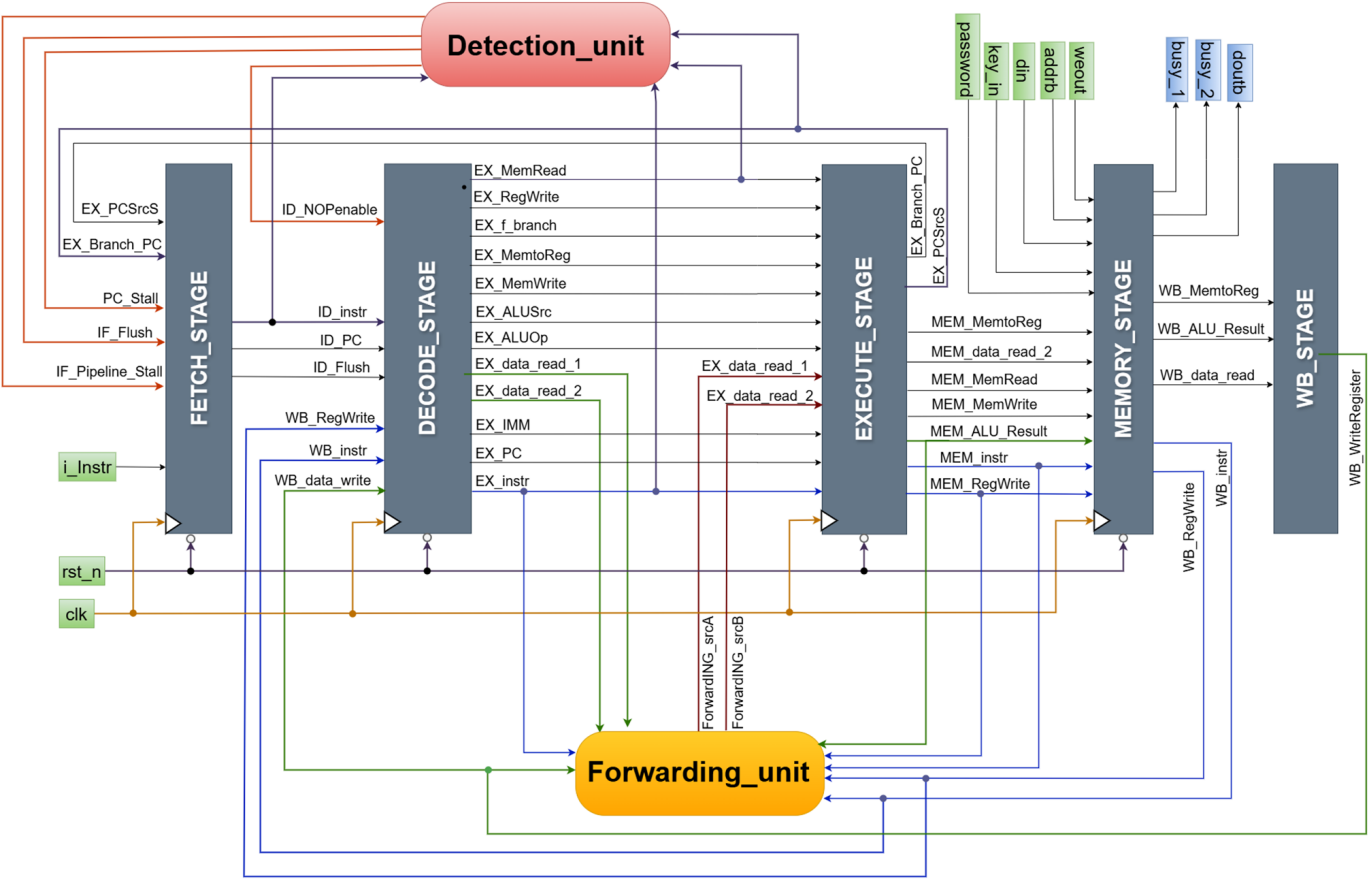
System block diagram. i.e. The signal names begin with an abbreviation of their originating stage.

**Fig. 7:**
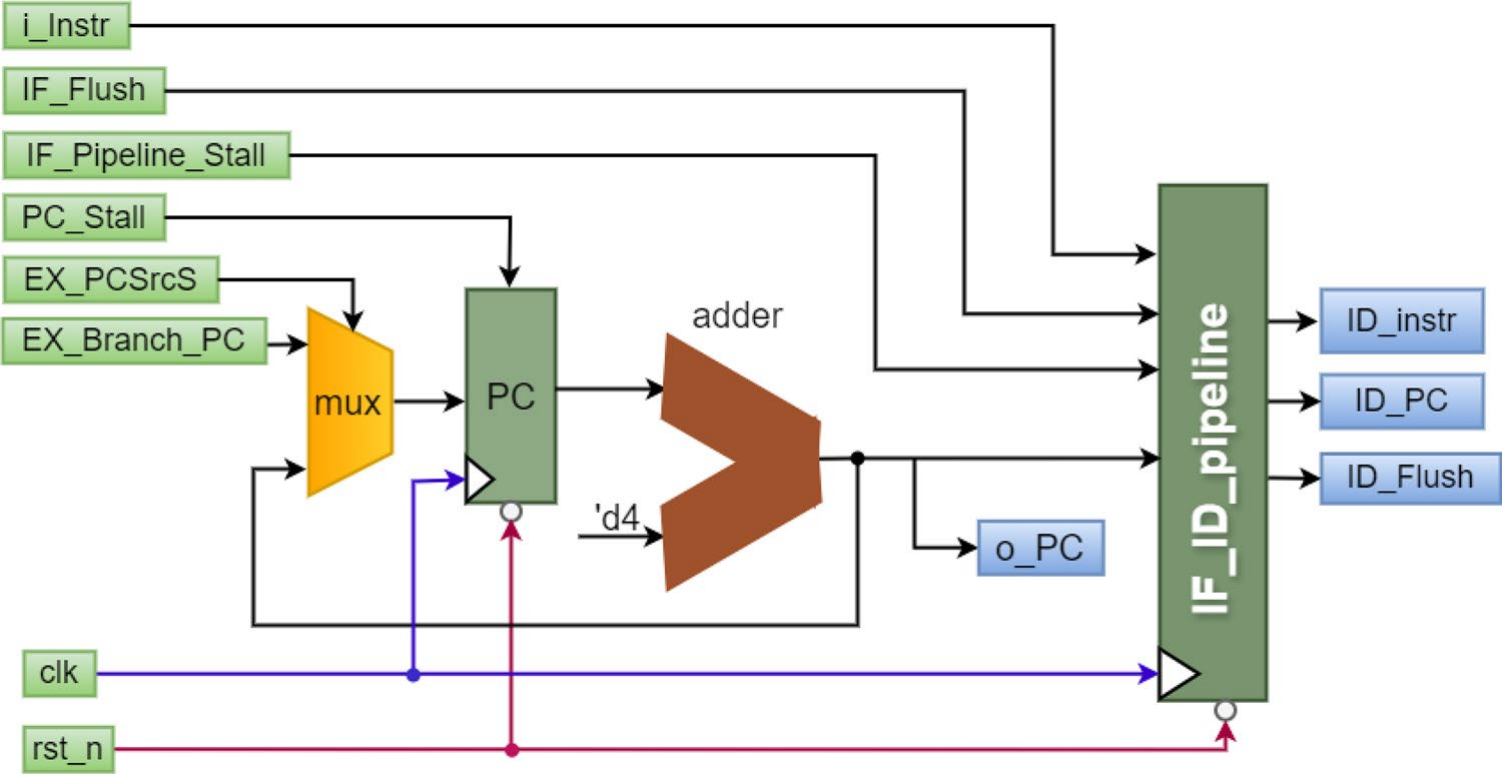
Block diagram of the implemented fetch stage.

The block diagram of the complete system is shown in Fig. 6. Firstly, the fetch stage reads the current instruction $i\_Instr$ with the address $o\_PC$ from an external instruction memory, as shown in Fig. 7. The adder prepares the address of the next inline instruction by adding 4 bytes (32 bits) to the current address. The multiplexer chooses and stores the address of the next instruction in the Program Counter (PC) register according to the $EX\_PCSrcS$ signal. This signal determines whether the next address is the inline or branch. The $EX\_Branch\_PC$ is the PC value for the branch instruction. In the case of a hazard, the signal $PC\_stall$ stalls the pipeline by deactivating the program counter to prevent the co-processor from proceeding to a new address. Also, it sets the $IF\_Flush$ signal that clears the control unit signals of the stalled instruction. The register between the fetch and decode stages, $IF\_ID\_Pipeline$, buffers the necessary signals from the fetch stage to the decode stage. It outputs the new program counter address $ID\_PC$ and instruction $ID\_instr$ when the stall $IF\_Pipeline\_Stall$ signal is disabled.

**Fig. 8:**
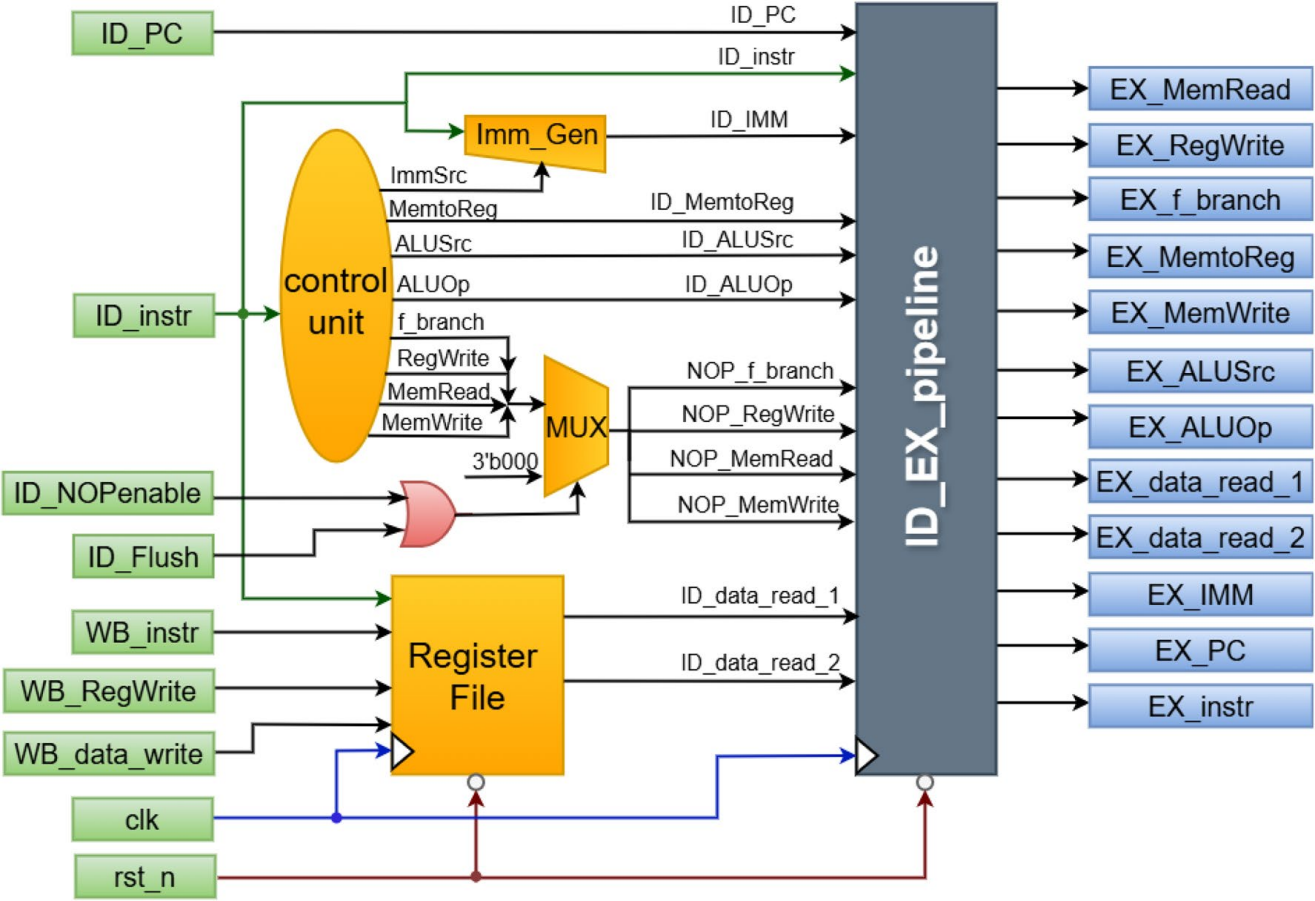
Block diagram of the implemented decode stage.

The next stage includes the control unit of the co-processor. The decode stage interprets the fetched instruction and generates the control signals for the subsequent stages, as shown in Fig. 8. The immediate generator $IMM\_Gen$ converts the immediate part of the instruction into a 32-bit format to be ready for the ALU. It also contains the register file that stores the operands of the instruction. The register file is implemented with an asynchronous read mode to allow data retrieval at any time, and a synchronous write mode with the negative edge of the clock cycle. It has 32 registers, each 32 bits wide. A multiplexer is used to select the right signal based on detected hazards. The register between the decode stage and execute stage $ID\_EX\_pipeline$ passes the essential signals to the execute stage. Table 2 demonstrates the decode stage signals.

**Table 2:** Decode stage signals definition.

Stage	Signal name	Description
Decode	ID_IMM	The extended signed data produced from the immediate value.
	ID_ALUSrc	Multiplexer selection signal determines whether the input to the
		ALU is from the register file or the sign extension.
	ID_ALUOp	control signal used to determine the ALU operation.
	NOP_f_branch	It is responsible for giving branch permission or not.
	NOP_RegWrite	The write enable signal of the register file.
	NOP_MemRead	The read enable signal of the Data Memory.
	NOP_MemWrite	The write enable signal of the Data Memory.
	ID_data_read_1	The data read from the first input address in the register file.
	ID_data_read_2	The data read from the second input address in the register file.
	ImmSrc	Determine how to generate the signed data from the immediate value.
	ID_MemtoReg	Multiplexer selection signal determines whether the returned data
		to the register file is the ALU output or the data from memory.
Decode &	ID_NOPenable	clear signal sets when there is a hazard and needs to
Detection_Unit		disable any action in the next stages.
	EX_RegWrite	The write enable signal of the register file.
	EX_f_branch	The branch-flag signal.
	EX_MemtoReg	The ID_MemtoReg signal.
Decode &	EX_MemRead	The read enable of the Data Memory.
Execute	EX_MemWrite	The write enable signal of the Data Memory.
	EX_ALUSrc	The ALU control signal.
	EX_IMM	The extended signed data.
	EX_instr	The instruction.
	WB_instr	Pipeline buffered version of the instruction in the Write-Back stage
		to give the address of the writing data.
Decode &	WB_RegWrite	Pipeline buffered version of the write enable of the register file in the
Write-Back		Write-Back stage.
	WB_data_write	output of the Write-Back stage that holds the data to be written in
		the register file.

**Fig. 9:**
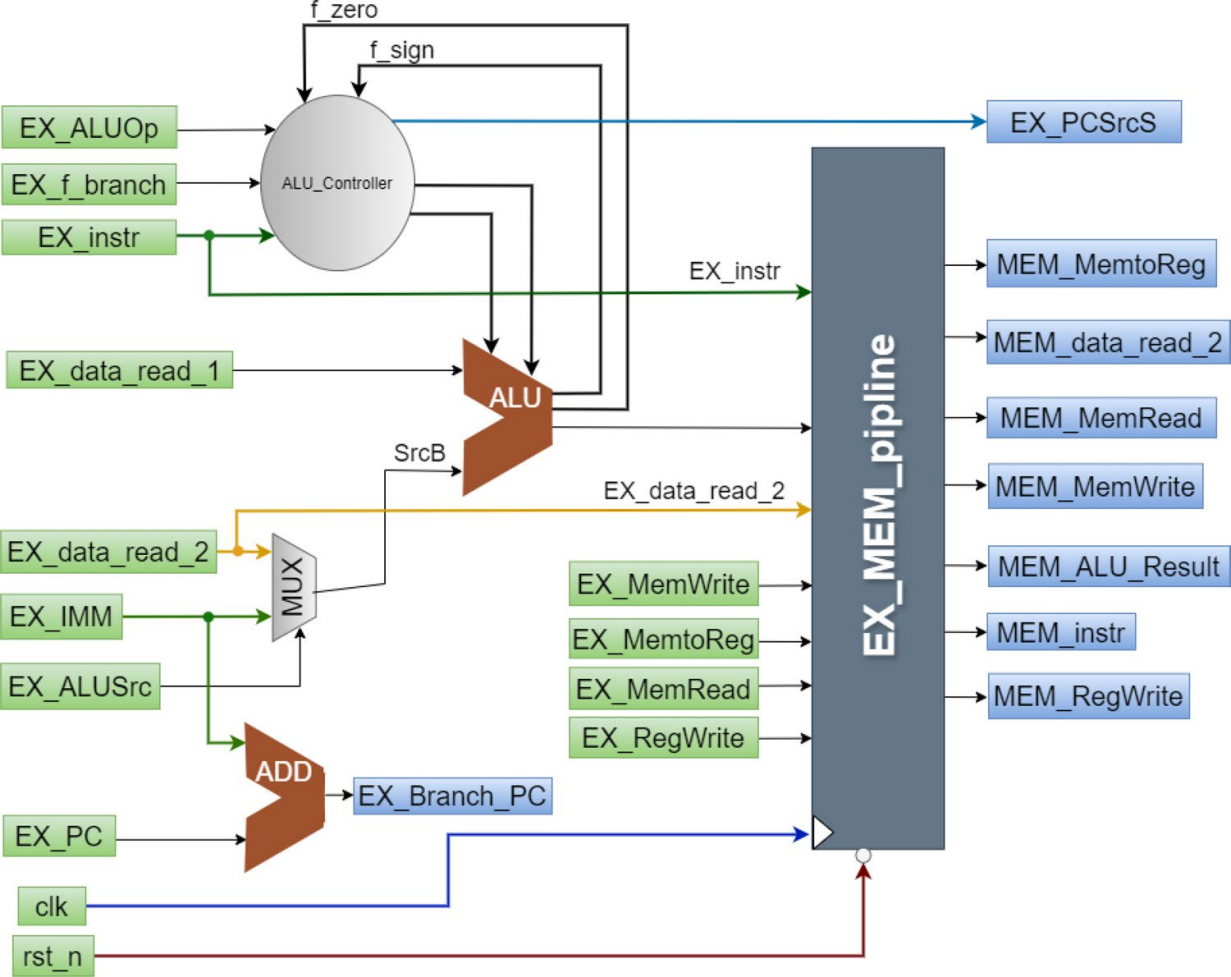
Block diagram of the implemented execution stage.

The main component of the execution stage is the arithmetic and logic unit (ALU) that performs the essential arithmetic and logical operations on two operands. The two operands are sourced from the register file or the forwarding unit, while the second operand *SRcB* involves the immediate data. The block diagram of the execution stage is illustrated in Fig. 9. A separate adder computes the new address for the program counter during branch instructions. The ALU controller manages the flags. The $F\_sign$ flag signal is triggered when the ALU operation is subtraction and its result is negative. The $f\_zero$ flag signal is set when the ALU result is zero.

The implementation of the memory stage was discussed in the previous subsections. The MEM_WB_Pipeline register buffers the required signals from the memory stage to the last stage, the write-back stage.

**Fig. 10:**
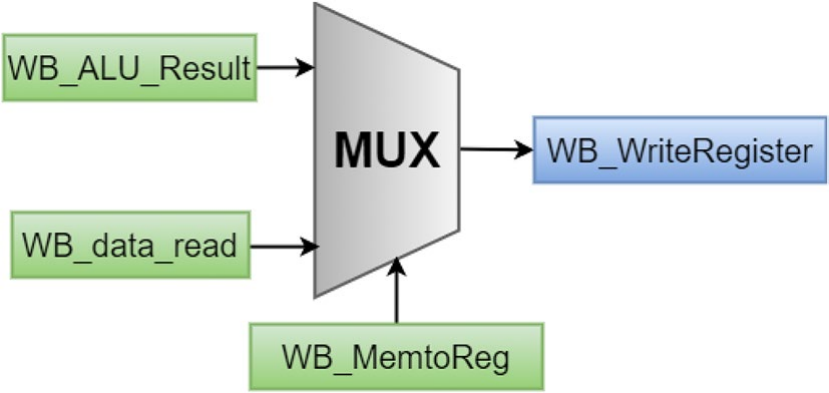
Block diagram of the implemented write-back stage.

The write-back stage updates the register file with the results of previous operations, memory access, or ALU execution. As illustrated in Fig. 10, it is the multiplexer that determines the source of the data to be written back to the register file.

**Fig. 11:**
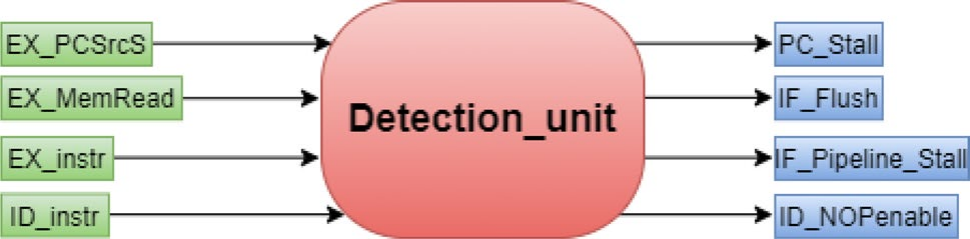
Inputs and outputs of the detection unit.

Management of hazards is crucial to optimize pipeline performance and maintain program execution integrity. Two blocks are implemented to manage hazards, detection, and forwarding. The inputs and outputs of the detection unit are depicted in a distinct diagram in Fig. 11. It is responsible for inserting stall clocks when it detects a data hazard caused by a load on the Read-After-Write (RAW) case or a control hazard caused by a branch instruction. When reading data from the memory, we recognize that RAW hazards are essential to accessing the most recent value. We use forwarding and stalling techniques to handle data and control hazards. Forwarding technique allows the result of an instruction to be used directly by subsequent instructions without waiting for it to be written back to the register file. This technique effectively reduces the delay caused by data dependencies, ensuring that the correct values are used in arithmetic and logical operations. Stalling involves pausing the pipeline until the data is available. The process ensures that subsequent instructions do not proceed until the required data is ready, preventing incorrect execution. While stalling introduces a delay, it is a straightforward method to manage data hazards in the load instructions.

**Fig. 12:**
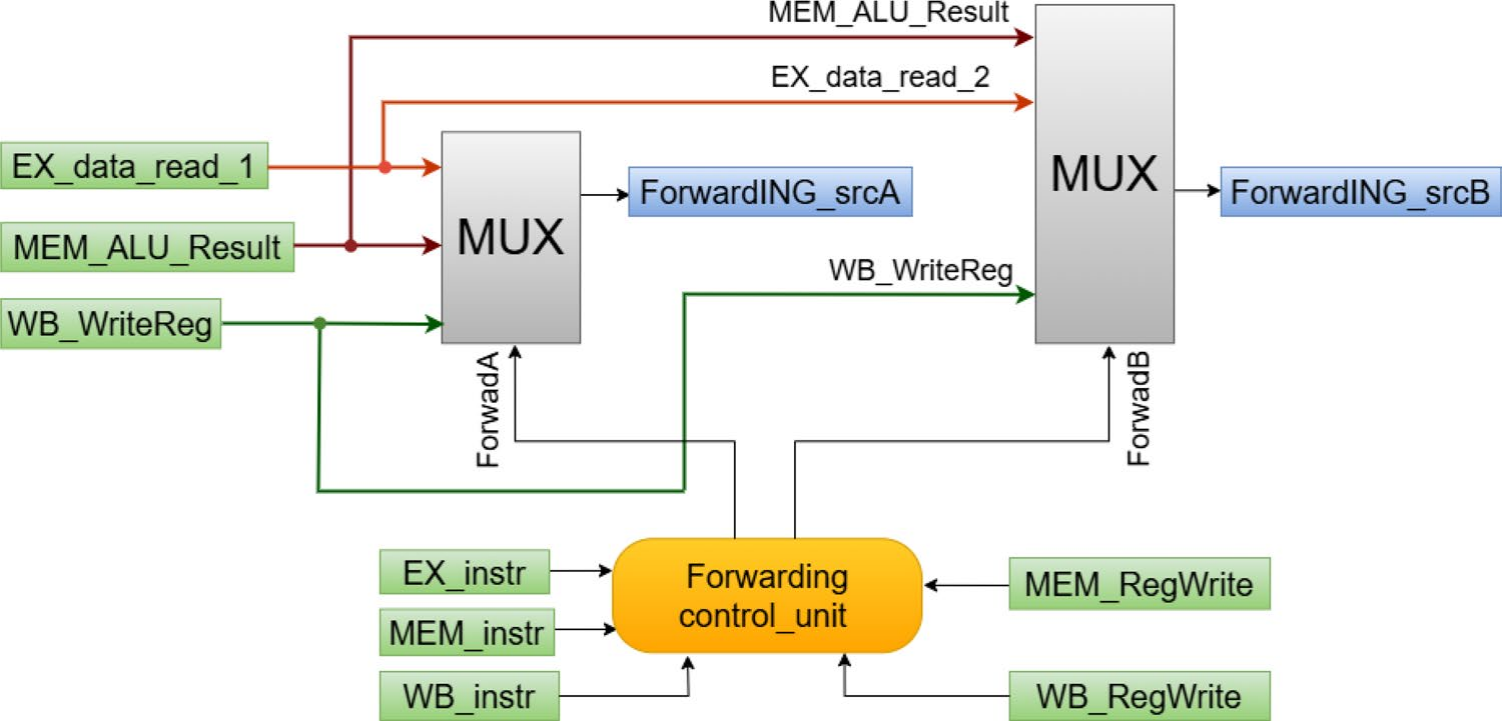
Block diagram of the implemented forwarding unit.

Figure 12 shows the detailed architecture of the forwarding block. The forwarding control unit determines the selection signals of the multiplexers. It decides whether each ALU operand should be sourced directly from the execution stage or forwarded from the memory or write-back stages. It handles the RAW data hazards by forwarding the correct data from the memory and write-back stages directly to the execution stage, ensuring that the ALU operates on the most up-to-date values. Each multiplexer selects the appropriate data source for one ALU operand according to the *ForwardINGsrcA* and *ForwardINGsrcB* selection signals.

### Cryptographic commands

Encryption, decryption, and hashing operations are our added cryptography commands. To execute these commands, we write the cryptography data in their assigned locations in the unified memory. The cryptography data is the input/output data files, the size of the input data, the cipher algorithm, and the user/cipher ID to get its key from the protected keys’ memory. The compiler should convert the cryptographic commands to the supported RISC-V instructions. The implemented cryptography ASIP supports only 21 RISC-V RI32 instructions, which will be stated later in this subsection. Then, a software assembler is used to convert the assembly instructions into the corresponding machine code.

Table 3 shows the required control commands and the associated RISC-V RV32I instructions that control the cryptographic operations according to Fig. 5. The check commands *CHK* verify the acknowledgment status using an instruction *LW* (load word) to load the value stored at the acknowledgment address into the register file. Then, a conditional branch instruction *BNE* (branch if not equal) checks the loaded value. The *TRIG* command starts the cipher operation by writing a value of one to the start register in the data memory. The instruction *SW* (store word) initiates the operation by signaling the DMA controller. Before executing these operations, it is necessary to calculate the effective addresses of the acknowledgment and start registers. This is typically done using the *ADDI* (add immediate) instruction to compute and store these addresses in the RISC-V register file. The base address of the cipher block input to these commands is to be mapped to data memory.

**Table 3:** Cryptographic commands.

Command	Instructions	Comment
CHK_AES -address ack_reg	LW rd, aes_base_addr, ack_reg_offset	rd $\xleftarrow {}$ M[base + offset]
	bne rd, 1, branch_offset	if (rd != 0) TRIG_AES;
		else goto : branch_offset;
TRIG_AES -address start_reg	sw 1 , start_reg_offset(aes_base_addr)	M[base + offset] $\xleftarrow {}$ 1
CHK_SHA -address ack_reg	LW rd, sha_base_addr, ack_reg_offset	rd $\xleftarrow {}$ M[base + offset]
	bne rd, 1, branch_offset	if (rd != 1) TRIG_SHA;
		else goto : branch_offset;
TRIG_SHA -address start_reg	sw 1 , start_reg_offset(sha_base_addr)	M[base + offset] $\xleftarrow {}$ 1

#### RISC-V RI32 supported instructions

Our cryptography ASIP supports eight R-type, nine I-type, one S-type, and three B-type instructions, as illustrated in Table 4. They support only 32-bit data size, which equal to the memory word. The R-type instructions support the basic arithmetic instructions, including addition *ADD*, subtraction *SUB*, logical shift left *SLL*, logical and arithmetic shift right *SRL* *SRA*. In addition to the logic instructions *OR*, *AND*, and *XOR*. Logic and arithmetic instructions are supported using an immediate value instead of the register *rs*2, which are *ADDI*, *SUBI*, *SLLI*, *XORI*, *SRLI*, *SRAI*, *ORI*, *ANDI*. The I-type load instruction *LW* loads the data from the memory address $(rs1 + immediate value)$ into the register *rd*. The S-type store instruction *SW* stores the data of the register *rs*2 in the memory address $(rs1 + immediate value)$. Three B-type instructions are implemented for branching. In the *beq*, branch if equal, and *bne*, and branch if not equal, instructions, the branch is taken when the registers *rs*1 and *rs*2 are equal or not equal, respectively. The branch is taken when the register *rs*1 is greater than *rs*2 in the *bgt*, branch if greater than, instruction.

**Table 4:** Supported instruction formats of the RI32 RISC-V^37^.

31 25	24 20	19 15	14 12	11 7	6 0	Type
func7	rs2	rs1	funct3	rd	opcode	R-Type
00000000	rs2	rs1	000	rd	1000000	ADD
01000000	rs2	rs1	000	rd	1000001	SUB
00000000	rs2	rs1	001	rd	1000000	SLL
00000000	rs2	rs1	100	rd	1000000	XOR
00000000	rs2	rs1	101	rd	1000000	SRL
01000000	rs2	rs1	101	rd	1000001	SRA
00000000	rs2	rs1	110	rd	1000000	OR
00000000	rs2	rs1	111	rd	1000000	AND

imm[11:0]		rs1	funct3	rd	opcode	I-Type
imm [11:0]		rs1	000	rd	0100000	ADDI
imm [11:0]		rs1	001	rd	0100000	SUBI
imm [11:0]		rs1	010	rd	0100000	SLLI
imm [11:0]		rs1	011	rd	0100000	XORI
imm [11:0]		rs1	100	rd	0100000	SRLI
imm [11:0]		rs1	101	rd	0100000	SRAI
imm [11:0]		rs1	110	rd	0100000	ORI
imm [11:0]		rs1	111	rd	0100000	ANDI
offset [11:0]		rs1	010	rd	0010000	LW

imm[11:5]	rs2	rs1	funct3	imm[4:0]	opcode	S-Type
offset[11:5]	rs2	rs1	010	offset[4:0]	0001000	SW

imm[12—10:5]	rs2	rs1	funct3	imm[4:1—11]	opcode	B-Type
offset[12—10:5]	rs2	rs1	001	offset[4:1—11]	0000100	beq
offset[12—10:5]	rs2	rs1	010	offset[4:1—11]	0000100	bne
offset[12—10:5]	rs2	rs1	100	offset[4:1—11]	0000100	bgt

#### Assembler

**Fig. 13:**
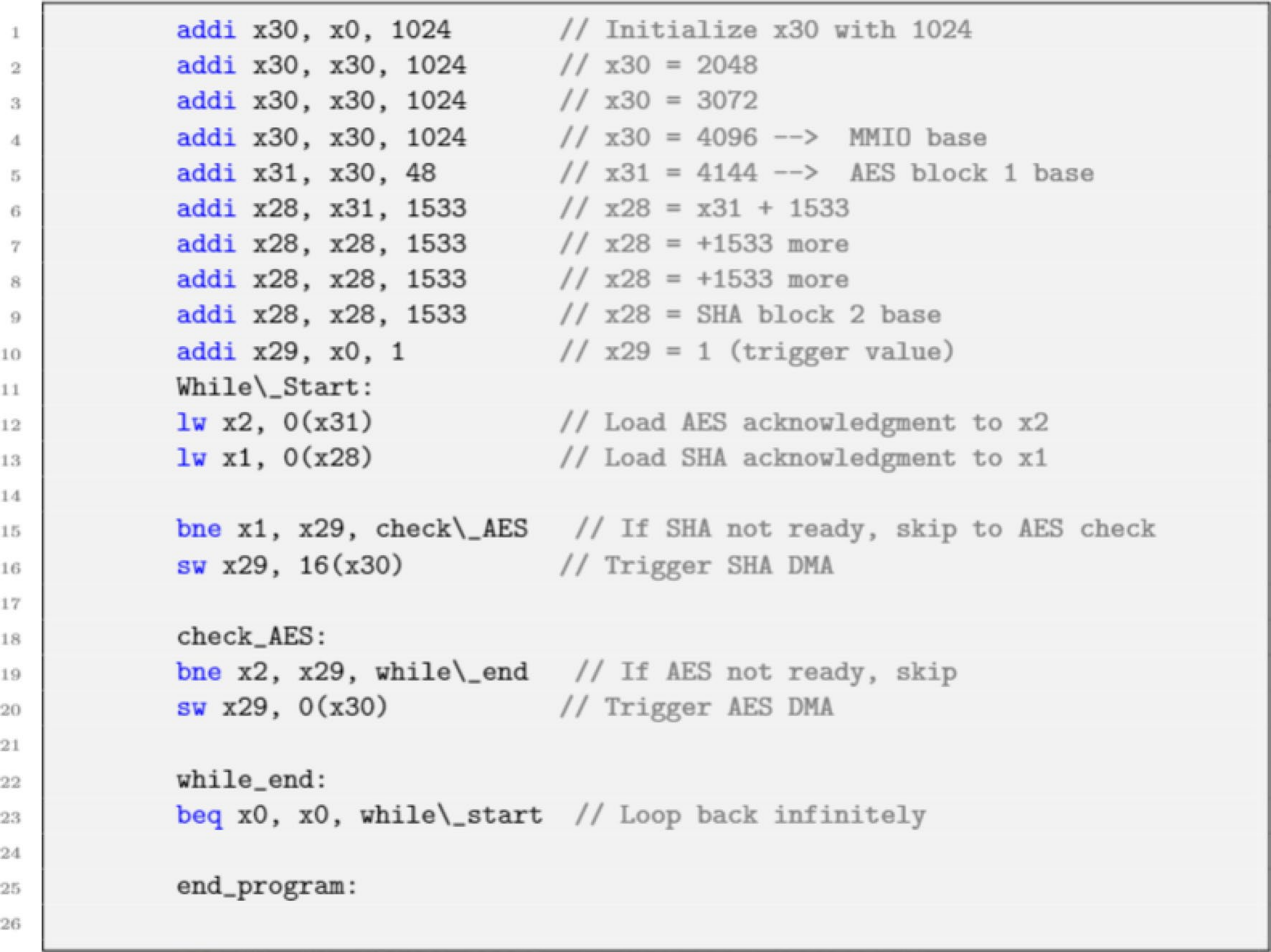
Assembly code of the co-processor’s main routine.

Figure 13 shows the assembly code designed for the proposed program that operates the cipher block on the co-processor. All address values used in the code are selected according to the memory layout illustrated in Fig. 3. The assembly routine starts by initializing register *x*30 with the base address of the memory-mapped I/O (MMIO) registers. This is achieved by incrementally adding 1024 four times to *x*30, resulting in a final value of 6806. Then, register *x*31 is assigned by the base address of the cipher block 1 memory, AES block, by adding 48 to the register *x*30. To calculate the base address of the cipher block 2 memory, SHA-256 block, four successive additions by 1533 are performed on *x*31. The result is stored in the register *x*28. Additionally, register *x*29 is set to 1, which will be used both as a trigger value and as a comparison constant.

The main execution loop begins by continuously reading the acknowledgment registers of both the AES and SHA blocks using the load instructions. These values are then compared with the expected ready signal (value of 1) stored in the register *x*29. If the SHA-256 block is ready, $x1 = 1$, the value 1 is written in the corresponding MMIO register to trigger the SHA DMA operation. Similarly, if the AES block is ready, $x2 = 1$, the same trigger value is written to the base address of the AES MMIO register to initiate the AES processing. This checking and triggering process loops indefinitely using branch instruction that jumps conditionally back to the start of the loop. Table 5 presents the instructions along with their corresponding machine codes for reference. Register *x*30 stores the base address of the IO register. Register *x*31 stores the base address of block 1 (AES), which is the AES data before processing. Then a four statements are used to add the depth of the AES block 1 to reach the base address of the AES block 2 and store it in the register *x*28.

**Table 5:** Instructions with its machine code.

Instruction	Machine code
addi x30, x0, 1024	40000f13
addi x30, x30, 1024	400f0f13
addi x30, x30, 1024	400f0f13
addi x30, x30, 1024	400f0f13
addi x31, x30 ,48	030f0f93
addi x28, x31 ,1533	5fdf8e13
addi x28, x28 ,1533	5fde0e13
addi x28, x28 ,1533	5fde0e13
addi x28, x28 ,1533	5fde0e13
addi x29, x0 ,1	00100e93
lw x2, 0(x31)	000fa103
lw x1, 0(x28)	000e2083
bne x1, x29, check_AES	01d09463
sw x29, 16(x30)	01df2823
bne x2, x29, while_end	01d11463
sw x29, 0(x30)	01df2023
beq x0, x0, while_start	fe0004e3
end_program	00000000

## Results and discussion

The security co-processor design is written using the Verilog hardware description language. It is synthesized and implemented on the Xilinx ZCU-102 FPGA board using the Vivado 2022.2 tool. The system is verified using standard benchmarks for AES-128 and SHA-256 operations. This section is divided into simulation and implementation results, as well as comparison with varied state-of-the-art systems.

### Implementation results

The performance of the proposed system, which includes a simple RISC-V implementation, AES-128, and SHA-256, is shown in Table 6. Performance metrics are area, delay, and power consumption. The throughput, power, and energy metrics of each cipher operation are calculated using Eqs. (1)-(4). The area after the implementation process is reported by the utilization and the number of configurable logic blocks (CLBs). Utilization includes the number of lookup tables (LUTs), flip-flops (registers), fast-carry logic (CARRY8), multiplexers (Muxes), 36 K-bit configurable synchronous random access memory (RAMB36E2) blocks, digital signal processing (DSP) blocks, and input/output blocks (IOBs). The maximum frequency of the proposed system is equivalent to the inverse of the worst path delay, which is equal to 15.6 ns with only 4.42 ns logic delay. The critical path (longest path) of the proposed design is the non-pipelined AES-128 datapath, which has 11 round stages. The implemented RISC-V core is synthesized without the AES-128 and SHA-256 accelerators; it has a frequency of 125 MHz. So, introducing internal pipelining into the AES datapath would increase the achievable clock frequency from 64 MHz to 125 MHz. However, this proposed architecture prefers to keep the cipher blocks simple and focus on the co-processor integration and data-transfer architecture.

1 $$\begin{aligned} Throughput= & \frac{ f \times \text {block size (bits)} }{\text {number of clock cycles/block} }, \end{aligned}$$

2 $$\begin{aligned} \text {Energy Consumption}= & \text {Latency} \times \text {Power Consumption}, \end{aligned}$$

3 $$\begin{aligned} \text {Energy/Operation}= & \frac{\text {number of clock cycles}/operation \times \text {Power Consumption}}{f}, \end{aligned}$$

4 $$\begin{aligned} \text {Power efficiency}= & \frac{\text {Throughput}}{\text {Power Consumption}}. \end{aligned}$$

The co-processor initiates the cipher operations, and then they work individually and in parallel. The core non-pipelined AES-128 and the SHA-256 take 1 and 68 clock cycles, respectively. Their input block size is 128 for the AES-128 and 512 for the SHA-256. So, the throughput of AES-128 and SHA-256 cipher operations is 8,192 and 482 Mbps, respectively. The computed cycle count is the core cycle count for each cipher operation, as it is mostly used in the state-of-the-art. However, the used RISC-V data bus is 32-bit, which adds 8 and 24 cycles for AES-128 and SHA-256, respectively. The 128-bit block size of the AES-128 takes 4 cycles for input deserialization and 4 cycles for output serialization. The 512-bit block size of the SHA-256 requires 16 cycles for deserialization and 8 cycles for serialization. The total number of clock cycles with the serializer and the deserializer blocks equals 9, and 92 for the AES-128 and SHA-256, respectively. This corresponds to a throughput of 910.2 Mbps for AES-128 and 356.2 Mbps for SHA-256. Using RISC-V with larger data bus sizes, 64 or 128 bits, will increase the total throughput of the cipher operations.

**Table 6:** FPGA performance after the implementation process for the proposed co-processor design on the ZCU102 FPGA kit.

Utilization	CLB LUTs	15964
	CLB registers	3945
	CARRY8	109
	Muxes	0
	Block RAMB36E2	9
	DSPs	0
	Bonded IOB	323
Data path delay (ns)	Total	15.6
	Logic	4.42
	Route	11.18
AES-128 cycle counts	Core	1
	Serializer/Deserializer	8
SHA-256 cycle counts	Core	68
	Serializer/Deserializer	24
Maximum frequency of the co-processor (MHz)		64
Throughput (Mbps)*	AES-128	8192
	SHA-256	482
On-chip power (W)	Total	1.575
	Dynamic	0.950
	Static	0.626

*Throughput (Mbps) = (*f* $\times$ block_size_bits) / core_cycles

To enhance clarity and facilitate understanding of the implementation details, the interface and data-flow information summarized in Table 7 is presented through the schematic representation shown in Fig. 6. This initial implementation was performed to validate the design on an FPGA with a wrapper interface to simplify the test process. The native RTL implementation exposes a relatively large number of low-level control and data ports, which can reduce readability and complicate top-level FPGA integration. Therefore, in practical deployment, the proposed design is packaged as an AXI4-Lite IP core. This approach significantly reduces the number of external ports by mapping internal control, status, and configuration signals to AXI-Lite registers, enabling standardized memory-mapped access and simplifying system integration with the FPGA platform

**Table 7:** Descriptions of the FPGA port signals.

Port name	Direction	Size (bits)	Description
clk	Input	1	System clock signal
rst_n	Input	1	Asynchronous active low reset signal
weout	Input	1	Write enable to load plain text into RISC-V data memory
addrb	Input	32	Address used for load and store from RISC-V data memory (plain & cypher text)
din	Input	32	Plain Text to be written into RISC-V data memory
password	Input	32	Access password for external users to insert or change keys
key_in	Input	128	Input keys (requires correct password and address)
doutb	Output	32	Read port output from data memory
busy_1	Output	1	Status flag indicating the AES block is busy
busy_2	Output	1	Status flag indicating the HASH block is busy

### Comparison with state-of-the-art

The proposed design is compared with several recent and diverse implementations of security ASIPs, co-processors, or SoCs. The ASIP and co-processor are custom processors; however, ASIP is designed for specific applications with custom instruction sets, while a co-processor handles special tasks as an extension to a main processor. Table 8 shows, for each design, the implemented security algorithms, architectural techniques, and the used technology. The metrics include area and frequency, as well as throughput and number of clock cycles for each security algorithm. The area is represented by the number of LUTs or gates for the combinational area and the number of Registers or Memory cells for the sequential area.

Although different architectures, the area represented by resource utilization and the number of clock cycles are independent of technology. However, the area is related to whether the core processor is built-in or not. The built-in processor does not appear in the FPGA resource utilization report. Also, the area increases with the number of supported security algorithms. The maximum frequency is affected by the technology. And the throughput, Eq. (1), depends on the frequency and the number of pipeline stages for each algorithm. It indicates a tradeoff between these two factors. As the number of pipeline stages, which is defined as the number of clock cycles, increases, the maximum frequency increases. However, it also increases the sequential area expressed as the number of registers and, therefore, dynamic power/energy consumption.

For the first row in Table 8, the crypto-coprocessor, as defined by its authors,^34^, is just a pipelined implementation of two security algorithms, AES-128 and SHA-3. They increase the frequency and throughput by increasing the number of pipeline stages. However, they have a huge sequential area. The implemented design uses 285 logic slices and 4 BRAMs. To express the total FPGA resource usage in a single unit, the paper converts BRAMs into “Equivalent Slices” using the assumption that one 32Kb BRAM is equivalent to 128 slices for Virtex-5/6/7 FPGAs. Therefore, the 4 BRAMs correspond to $4 \times 128 = 512$ equivalent slices. Adding these to the 285 actual logic slices gives a total of $285 + 512 = 797$ equivalent slices. It should be noted that they don’t have an integrated processor. So, it achieves maximum throughput and frequency for the specified algorithms. However, they need a system-level approach to coordinate operations and data management, which elevates software complexity.

The next rows in Table 8 present the state-of-the-art SoCs. Two architectural designs are employed: an FPGA-based soft/hard core processor in Refs. 32 and 33, and a combination of the open-source RISC-V processor with an implemented crypto processor in Refs. 28 and 29. These SoC designs maintain the integrity of the core processor’s ISA. However, they have resource area overhead resulting from the unused or redundant control logic and structures (e.g. two fetch/decode units).

Then, the ASIP architectures are presented in the table. Its custom ISA provides hardware efficiency tailored to the cipher algorithms. Its close integration within the processor execution stage reduces the latency and the control overhead. However, this architecture compromises compatibility and requires modifications to the compiler. Furthermore, this approach introduces additional complexity to the instruction set architecture, thereby increasing decoder logic and potentially complicating the verification process. Also, it has limited flexibility, as introducing a new cryptographic algorithm requires additional opcodes and modifications to the processor’s datapath, thereby reducing its scalability. The area of the different designs is dependent on the supported cipher blocks and the number of pipeline stages, which affect the sequential area. It should be noted that Le et al. in Ref. 30 calculate the throughput by number of cycles and Cycle/Byte, so they (Cycle/Byte) value is inverted and multiplied by the frequency and by 8 (as a byte equals 8 bits) to use the same equation, Eq. (1), for all reference designs.

The proposed MMIO/DMA-Driven Co-Processor occupies a unique position within this spectrum, emphasizing scalability, adherence to RISC-V standards, and controlled complexity. It is compatible with the standard RISC-V. New cipher blocks are incorporated as memory-mapped peripherals with a generic wrapper (serializer/deserializer), eliminating the need for modifications to the processor pipeline or control logic. It maintains separation of the responsibilities as the RISC-V core manages control flow and sequencing through straightforward load and store operations, whereas the DMA oversees high-bandwidth data transfer, thereby preventing pipeline delays. Furthermore, it enables the simultaneous execution of independent cipher blocks with dedicated memory regions. Despite this, it has an independent Latency overhead for MMIO access, and the serializer/deserializer stages impose a fixed overhead (e.g. 4+4 cycles for AES-128 and 8+16 cycles for SHA-256) relative to a specialized ISA that could supply data directly to the ALU.

The performance of various architectural designs, including the proposed one, can be observed in the throughput figure. Design without a processor, such as Ref. 34, has a relatively high throughput for the SHA-256, 5.6 times that of the proposed design. It utilizes purely optimized hardware pipelines, lacking system-level optimization. However, the non-pipelined AES-128, although it decreases the system clock cycle, has 3.4 times higher throughput. Custom ISA designs such as Ref. 30 offer an optimal balance between performance and software integration, although they often lose scalability and standardization. Compared with the proposed design, it has 3.3 times the frequency, with 11.2 times, and 1703 times less throughput for the SHA and AES, respectively. They increase the number of pipeline stages to increase the frequency at the cost of throughput. On the other hand, the SoC design benefits from the embedded processor speed and efficiency; however, this embedded processor has a large area according to its general-purpose nature. The AES results in Ref. 32 have the same comparison concept for frequency, pipeline stages, and throughput. They increase the frequency by large pipeline stages, but the proposed AES-128 has a better throughput by 27.8 times. However, the SHA results in Refs. 32 and 33 maintain high throughput with relatively high frequency, which expresses their efficient hardware optimization of the SHA algorithm. The work in Ref. 29 has almost the same frequency as the proposed design, with 10 times less throughput. It employs a pipelined AES core that can accept a new block every clock cycle. Moreover, their reported LUTs area is larger by 40% than the proposed design; noted that they support AES only and didn’t report their standard RISC-V processor area. Also, they have a very large number of clock cycles for initialization and software, which further decreases the total throughput to 89.4 Mbps. On the other hand, including the serializer/deserializer clock cycles for our proposed design gives a throughput of 910.2 Mbps for the AES-128 operation, around 10 times^29^. The proposed design intentionally balances latency and frequency within a single-block system to ensure strong compliance with standards, enhanced flexibility, and support for parallel execution. These considerations are all essential for a scalable security co-processor designed for use in IoT applications.

**Table 8:** Comparison between the proposed framework and state-of-the-art.

Design	Cipher blocks	Architectural techniques	Tech.	Area	Freq. (MHz)	Cycle counts	Throughput (Mbps)
^34^, 2020	AES-128, SHA-3	Pipelined, no processor	28 nm Virtex-7 FPGA	285 slices + 512 BRAM eq slices =797 slices	377	20	AES 2412 SHA 2713
SoC^32^, 2020	AES-128, ECC-163, SHA-256	MicroBlaze softcore	28 nm Artix-7 FPGA	17.2k LUTs 208 LUTRAM 2 BRAM	150	AES 65, ECC 55k, SHA 65	AES 295, ECC 0.44, SHA 590
SoC^33^, 2020	RSA-1024, SHA-256	build-in ARM hardcore	28 nm Cyclone-V FPGA	20k Logic elements	RSA 50 SHA 100	-	RSA 0.025 SHA 700
SoC^28^, 2021	AES, ECC, SHA-256	Pipelined, two processors include RISC-V, Custom ISA	28 nm ASIC	490k gates, 10k Mem.	197	-	-
SoC^29^, 2022	AES-128	two processors include RISC-V, MMIO	28 nm Artix-7 FPGA	22k LUTs, 843 MUXs 12k registers	65	83 init., 189 S/W, 10 H/W	832 (w/o init & S/W cost)
ASIP^10^, 2012	RSA-1024	3-stages pipelined RISC, Custom ISA	65 nm Virtex-5 FPGA	23k LUTs	200	1,356,384	0.151
ASIP^26^, 2016	SHA-3	Pipelined, MIPS-based, Custom ISA	40 nm Virtex-6 FPGA	7.65k slices 1.8kB Memory	200	178	9
ASIP^14^, 2016	MD5, SHA-1,-3, ...	7 pipeline stages, Custom ISA	65 nm ASIC	0.28 $mm^2$ 4.5kB memory, 52k logic gates	1000	164	15800
ASIP^27^, 2019	IDEA	Single-cycle, MIPS-based, Custom ISA	40 nm Virtex-6 FPGA	39k LUTs 39.5k Reg.	19.3	422	5.6
ASIP^15^, 2022	SHA-3	single cycle, Custom ISA	65 nm Virtex-5	2.1k LUT , 639 Reg.	213.4	113,622	3
ASIP^30^, 2024	SHA-256, AES128, ...	5-stages pipelined RISC-V, Custom ISA	16 nm ZCU102 FPGA	35k LUTs, 29.6k Registers 16 BRAM	210	SHA 2495 AES 5590	SHA 43.1 AES 4.81
Our Co- Processor	SHA-256, AES-128, scalable...	5-stages pipelined RISC-V, MMIO	16 nm ZCU102 FPGA	16k LUTs 4k Registers 109 CARRY8 9 BRAM	64	SHA 68, AES 1*	SHA 482 AES 8192**

*Cycle count = core_cycles

**Throughput (Mbps) = (*f* $\times$ block_size_bits)/core_cycles

The distinctive aspect of our security co-processor is the implementation of a simple version of the RISC-V processor with part of its standard instructions, Table 4. The proposed design features a scalable and modular structure and supports the concurrent execution of both AES-128 and SHA-256. Our design also supports easy integration of additional cipher blocks and parallel execution with clear signal interfacing (start/done) for synchronization and debugging. The use of a RISC-V processor combined with MMIO provides a lightweight, open, and scalable architecture compared to ASIPs or tightly coupled ISA designs^33^. MMIO enables several features, such as low-overhead interfacing with cryptographic blocks, enhanced control and debugging support, and seamless communication with system-level components.

#### Power/energy efficiency

The proposed design has 1.575 total power consumption. It improves power consumption by 63% compared to the recent state-of-the-art design in Ref. 30, which has a total power consumption of 4.22 W. Table 9 compares the power and energy of different state-of-the-art cryptographic implementations across FPGA and ASIC platforms. Earlier FPGA-based designs^34^ achieve very low latency through high operating frequencies, but this comes at the cost of high total power consumption, resulting in moderate energy per operation. ASIC-based SoC implementations^28^ demonstrate improved energy efficiency, particularly for AES-128, highlighting the advantage of custom silicon, although detailed power and latency figures are not fully reported. The recent ASIP design^30^ shows relatively low dynamic power but suffers from very large latency, leading to significantly higher energy consumption. In contrast, the proposed co-processor achieves a favorable balance between power and latency: despite operating at a lower frequency, its substantially reduced cycle count results in competitive energy consumption for SHA2-256 and notably lower energy for AES-128 compared to prior FPGA-based works, demonstrating the effectiveness of the proposed architecture.

**Table 9:** Comparative power/energy with state-of-the-art.

Design	Kit/Tech.	Cipher algorithm	Power(mW)		Clock	Freq.	Latency	Energy
			Dynamic	Total	cycles	(MHz)	($\mu$s)	(nJ)
^34^, 2020	28 nm	SHA-256	NA	790	20	377	0.0531	41.91
	Virtex-7	AES-128	NA	1070				56.76
	FPGA							
SoC^28^, 2021	28 nm	SHA2-256	NA	NA	NA	197	NA	27.13
	ASIC	AES-128	NA	NA	NA		NA	3.79
ASIP^30^, 2024	16 nm	SHA2-256	36	NA	2495	210	11.88	428
	ZCU-102	AES-128	18	NA	5590		26.62	479
	FPGA							
Our Co-Processor	16 nm	SHA2-256	41.87	NA	92	64	1.44	60.30
	ZCU-102	AES-128	102.5	NA	9**		0.141**	14.45**
	FPGA	Total on-chip	950*	1575				

NA: Not Available. *: The total dynamic power 41.87 + 102.5 + 805.63 (RISCV-overhead) = 950 mW. ** Depend on security algorithm.

#### Hardware gains over software

The performance of the proposed cryptographic co-processor is compared with CPU-only software implementations of AES-128 and SHA-256 on ARM Cortex-A53 and RISC-V processors. The two state-of-the-art baseline software platforms are the ARM Cortex A53^38^ and the RISC-V processor^39^. Table 10 compares throughput and power efficiency for different software processors and the proposed hardware design. For the ARM Cortex A53^38^, the throughput is calculated from the reported execution time of 8,192 test cases. The reported execution time is equal to 241.8 ms. The throughput equals the number of clock cycles (128 bits for the AES-128) multiplied by the number of test cases (8,192) and divided by the execution time.

The throughput improvements are 4.5 times for the AES-128 and 14.3 times for the SHA-256 over the RISC-V software baseline^39^. The total power consumption of the proposed system is 1.575 watts. The proposed co-processor hardware implementation has 525 times higher power efficiency (Eff.) compared to the ARM Cortex-A53 with respect to the AES software implementation. This result demonstrates the benefits of hardware implementation for energy-constrained IoT systems.

**Table 10:** Comparison of the proposed co-processor with CPU-only software implementations.

Algorithm	Metric	Cycle counts	Throughput (Mbps)	Frequency (MHz)	Power eff. (Mbps/W)
AES-128	Software only (ARM Cortex-A53)^38^	NA	4.3	200	1.1
	Software only (RISC-V)^39^	314	203	500	–
	Proposed Co-Processor	9*	910.2**	64	577.9
SHA-256	Software only (RISC-V)^39^	5049	25	500	–
	Proposed Co-Processor	92*	356.2**	64	226.2

*Cycle count = total_cycles = core cycles + serializer + deserializer. **Throughput (Mbps) = (*f* $\times$ block_size_bits)/total_cycles.

### Scalability

The proposed design is inherently scalable, allowing seamless integration of additional cipher blocks into the co-processor architecture. This is enabled by a centralized DMA unit that manages data transfers through a single memory interface using the memory-mapped I/O (MMIO) technique. Using the MMIO scheme, all cryptographic blocks are exposed to the RISC-V core through a uniform set of configuration and status registers. This register-based interface is independent of the specific algorithm, making the control interface generic and allowing new cipher blocks to be added without modifying the processor pipeline or the system bus. Data transfers to and from these blocks are handled by a centralized DMA unit that manages data movement through a single 32-bit memory interface.

On the control side, each peripheral (e.g. AES-128, SHA-256, or a future cipher block) is exposed to the RISC-V core via memory-mapped configuration and status registers. Adding a new block, therefore, only requires instantiating a new block-specific register set and connecting it to the MMIO address space. The RISC-V pipeline, DMA logic, and global interconnect remain unchanged. This uniform register interface makes the control path structurally scalable with respect to the number and type of cryptographic blocks.

On the data side, the DMA engine uses a 32-bit data bus to transfer data between the system data memory and the peripherals. Since AES-128 and SHA-256 require internal data widths larger than 32 bits, serializer and deserializer units are used at the input and output of each block to adapt between the 32-bit DMA bus and the wider internal datapaths. Each cipher block is connected to its own true dual-port BRAM. One port is used by the DMA to read and write 32-bit words, while the other port is used by the corresponding cipher core (and, when needed, the RISC-V core). This organization allows multiple peripherals to access their local memories in parallel, enabling concurrent operation of the active blocks.

Let block *i* process $b_i$ bits per operation in $C_i$ clock cycles at a clock frequency $f_{\text {clk}}$. Neglecting memory contention, the peak core-side throughput of block *i* is

5 $$\begin{aligned} Throughput_i^{\text {core}} = \frac{b_i \cdot f_{\text {clk}}}{C_i} \quad [\text {bits/s}]. \end{aligned}$$

If *N* cipher blocks are active in parallel, the ideal aggregate throughput offered by all cores is

6 $$\begin{aligned} Throughput_{\text {total}} = \sum _{i=1}^{N} Throughput_i^{\text {core}}. \end{aligned}$$

However, the DMA and memory subsystem impose a global bandwidth limit. The maximum theoretical memory bandwidth is

7 $$\begin{aligned} B_{\text {mem}} = \text {data bus width} \cdot f_{\text {clk}} \quad [\text {bits/s}]. \end{aligned}$$

For each operation of block *i*, let $R_i$ and $W_i$ denote the total number of bits read from and written to memory (e.g. plaintext/ciphertext, keys, and intermediate values). The effective throughput of block *i* cannot exceed the rate at which the DMA can supply and store this data.

8 $$\begin{aligned} Throughput_i^{\text {mem}} \le \frac{B_{\text {mem}}}{R_i + W_i}. \end{aligned}$$

When several blocks are enabled, the DMA arbitrates their requests and time-multiplexes the 32-bit bus across the different local BRAMs. As a result, the total system throughput is bounded by Eq. (9) with additional small overheads from arbitration and scheduling.

9 $$\begin{aligned} Throughput_{\text {total}} \le \min \!\left( Throughput_{\text {total}}^{\text {core}},\, B_{\text {mem}}\right) . \end{aligned}$$

The proposed design has two blocks (AES-128 and SHA-256). When more cipher blocks are added, the same MMIO and DMA infrastructure can be reused without modification. The aggregate throughput can increase approximately linearly with the number of active blocks until the shared DMA bus and BRAM ports become the dominant bottlenecks. Beyond that point, scalability is limited by memory bandwidth and DMA rather than by the RISC-V core or the cipher datapaths.

### Simulation results

**Fig. 14:**
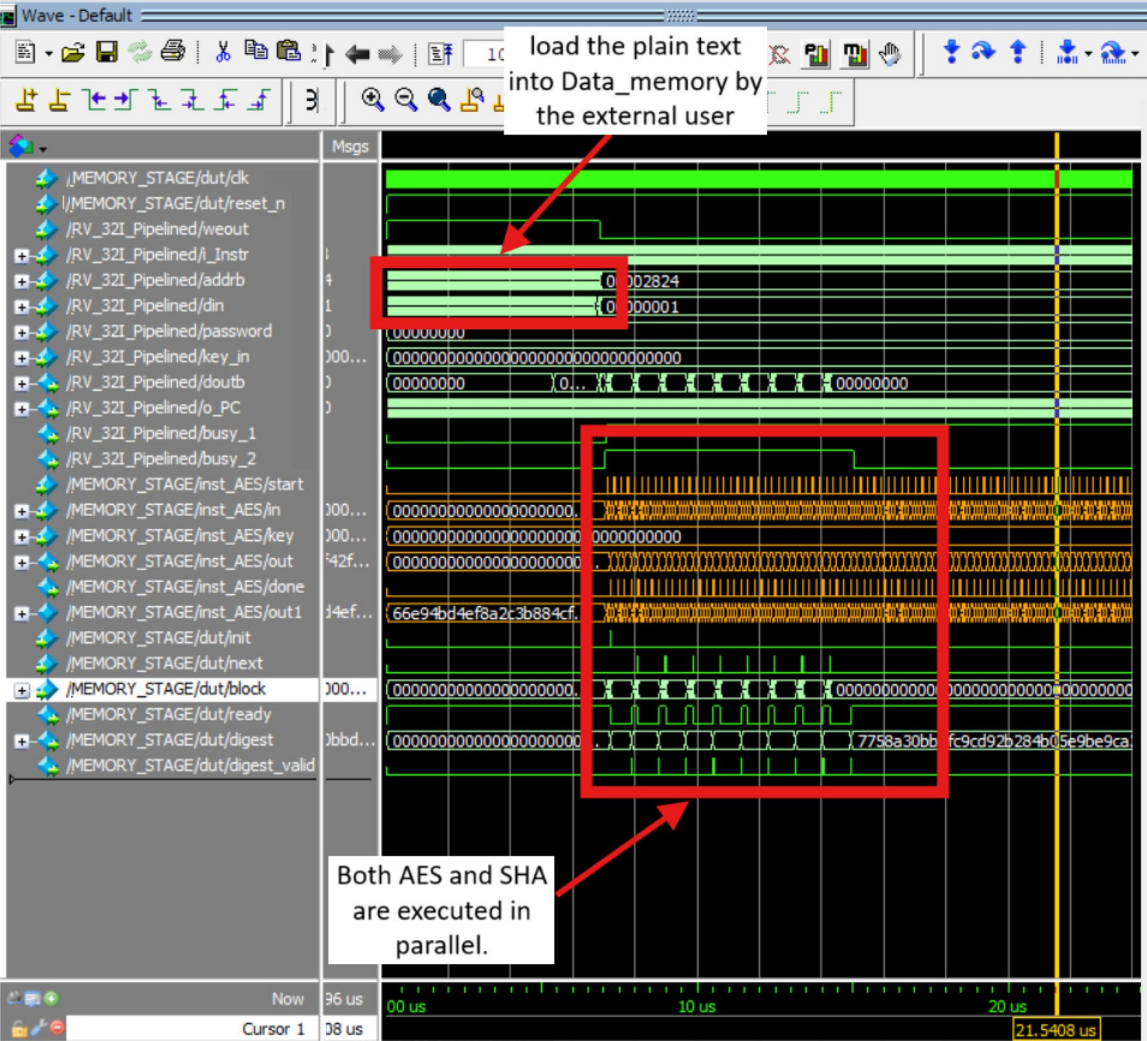
A simulation waveform that shows the parallel execution of the AES and SHA instruction sets.

Figure 14 presents a screenshot of the simulation waveform, which shows the parallel execution of the AES-128 and SHA-256 instruction sets. This capability to execute cryptographic operations concurrently is a critical feature in our proposed co-processor design, as it significantly enhances computational throughput, reduces latency, and enables more efficient utilization of hardware resources.

**Fig. 15:**
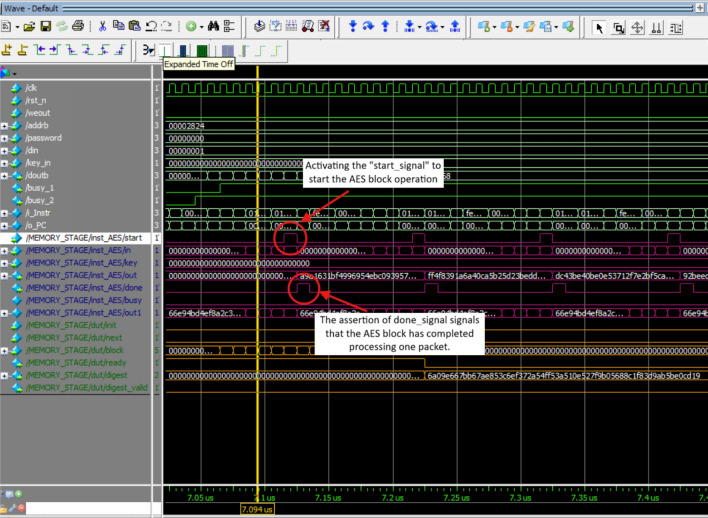
A simulation waveform that shows the execution of the AES-128 encryption with detailed control signal triggers.

Figure 15 shows the simulation waveform of the AES-128 encryption process. The waveform captures the activation of the *start_signal*, which initiates the AES block operation following instruction decoding, and the assertion of the *done* signal upon completion of the encryption. Incorporating such control signals into the design not only facilitates clear sequencing and coordination of cryptographic operations but also enhances the scalability of the co-processor architecture. By integrating these control signals as flags within control registers, the design supports efficient monitoring and debugging, which is essential for reliable integration, testing, and future extension of the cryptographic subsystem

**Fig. 16:**
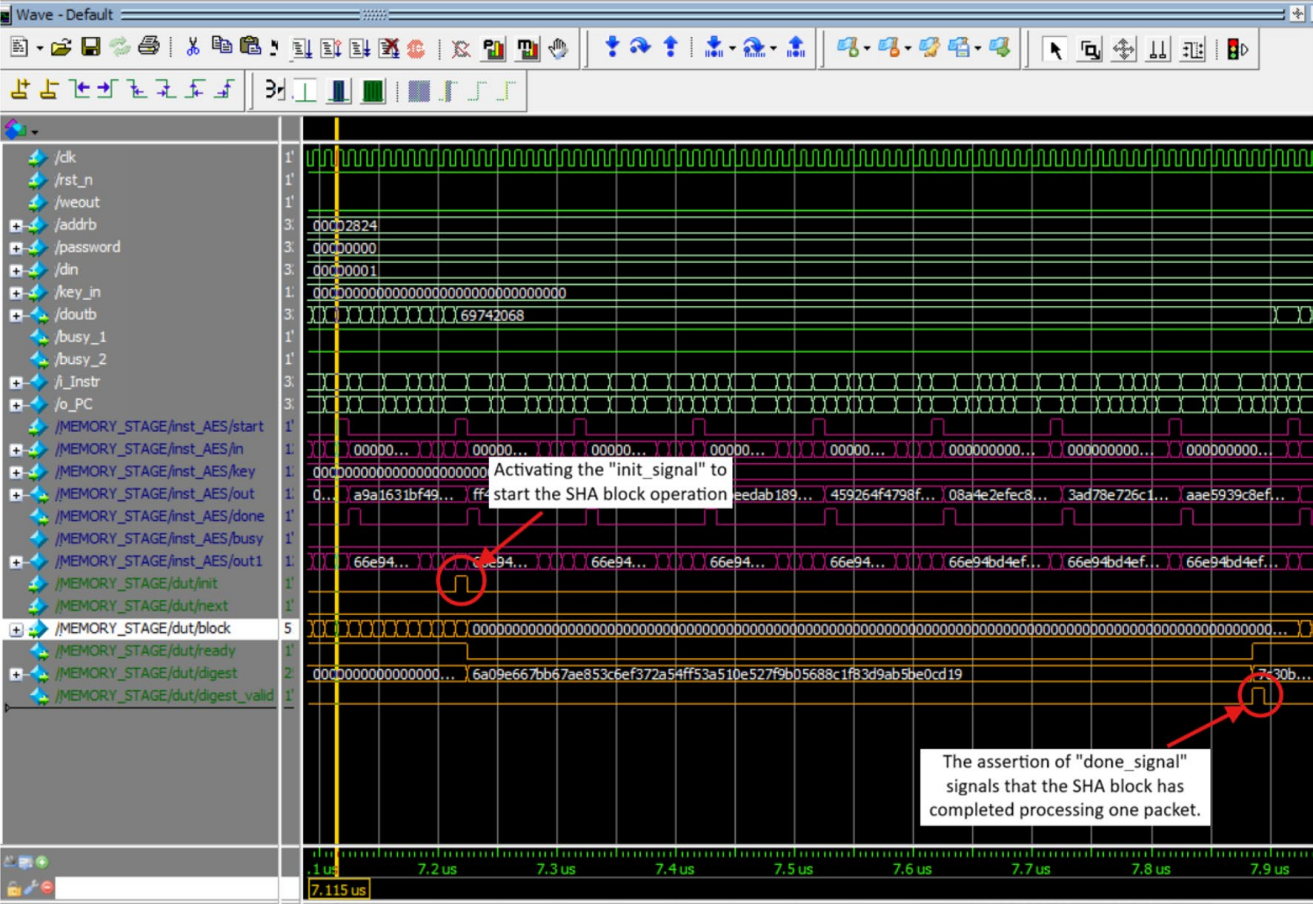
A simulation waveform that shows the SHA-256 hashing operation with detailed control signal triggers.

Figure 16 shows the simulation of the SHA-256 hashing operation. The waveform shows the activation of the $init\_signal$ trigger to start the hashing operation and it also shows the generation of the done signal that indicates the completion of the hashing task. Similar to AES operation, incorporating such control signals into the design help designers better debug and test their designs.

## Conclusion

This paper presented a novel cryptographic co-processor architecture based on RISC-V that successfully addresses the critical challenges of achieving high performance while maintaining energy efficiency and architectural flexibility in IoT devices. The experimental results show throughput rates of 8,192 Mbps for AES-128 and 482 Mbps for SHA-256 operations at a low operating frequency of 64 MHz. Our non-pipelined AES-128 datapath forms the overall frequency. It prioritizes architectural simplicity, moderate resource usage, and a generic RISC-V + MMIO/DMA integration framework over maximum possible operating frequency. Introducing additional pipeline stages inside the AES datapath would increase the maximum frequency. However, this performance represents a substantial improvement in energy efficiency compared to existing solutions in IoT applications, which typically require significantly higher clock frequencies to achieve comparable throughput.

Unlike previous work that targets fixed algorithm sets, our design supports the concurrent execution of multiple cryptographic operations and provides a generic interface for easy integration of additional cipher blocks. Resource utilization analysis demonstrates the efficiency of our approach, requiring only 15964 LUTs, 3945 registers, and 9 block RAMs. The integration of memory-mapped I/O and DMA modules ensured efficient data handling without introducing pipeline delays. We plan to extend our work by expanding the cryptographic algorithm support to include other cryptographic operations, exploring advanced optimization techniques for further power reduction, and investigating the integration of other standard interfaces to be compatible with more IoT frameworks.

## Data Availability

No datasets were generated or analysed during the current study.
